# Rice osa-miR171c Mediates Phase Change from Vegetative to Reproductive Development and Shoot Apical Meristem Maintenance by Repressing Four *OsHAM* Transcription Factors

**DOI:** 10.1371/journal.pone.0125833

**Published:** 2015-05-29

**Authors:** Tian Fan, Xiumei Li, Wu Yang, Kuaifei Xia, Jie Ouyang, Mingyong Zhang

**Affiliations:** 1 Key Laboratory of South China Agricultural Plant Molecular Analysis and Genetic Improvement & Guangdong Provincial Key Laboratory of Applied Botany, South China Botanical Garden, Chinese Academy of Sciences, Guangzhou, China; 2 University of Chinese Academy of Sciences, Beijing, China; 3 Key Laboratory of Plant Resources Conservation and Sustainable Utilization, South China Botanical Garden, Chinese Academy of Sciences, Guangzhou, China; 4 Rice Institute, Chongqing Academy of Agricultural Sciences, Chongqing, China; Institute of Botany, Chinese Academy of Sciences, CHINA

## Abstract

Phase change from vegetative to reproductive development is one of the critical developmental steps in plants, and it is regulated by both environmental and endogenous factors. The maintenance of shoot apical meristem (SAM) identity, miRNAs and flowering integrators are involved in this phase change process. Here, we report that the miRNA osa-miR171c targets four *GRAS* (*GAI-RGA-SCR*) plant-specific transcription factors (*OsHAM1*, *OsHAM2*, *OsHAM3*, and *OsHAM4*) to control the floral transition and maintenance of SAM indeterminacy in rice (*Oryza sativa*). We characterized a rice T-DNA insertion *delayed heading* (*dh*) mutant, where the expression of *OsMIR171c* gene is up-regulated. This mutant showed pleiotropic phenotypic defects, including especially prolonged vegetative phase, delayed heading date, and bigger shoot apex. Parallel expression analysis showed that osa-miR171c controlled the expression change of four *OsHAMs* in the shoot apex during floral transition, and responded to light. In the *dh* mutant, the expression of the juvenile-adult phase change negative regulator osa-miR156 was up-regulated, expression of the flowering integrators *Hd3a* and *RFT1* was inhibited, and expression of *FON4* negative regulators involved in the maintenance of SAM indeterminacy was also inhibited. From these data, we propose that the inhibition of osa-miR171c-mediated *OsHAM* transcription factors regulates the phase transition from vegetative to reproductive development by maintaining SAM indeterminacy and inhibiting flowering integrators.

## Introduction

microRNAs (miRNAs) regulate gene expression by sequence-specific cleavage or translational repression of cognate mRNAs in plants and animals [1,2]. They are involved in most of the essential physiological processes in plants, including organ development, hormone signalling, and stress response [3–6]. It is particularly worth mentioning that they have diverse roles in plant development, such as phase transition, flowering, leaf morphogenesis, meristem identity, and other aspects of plant development [7–9].

Higher plants pass through a series of developmental states to complete their life cycles. During post-embryonic life, juvenile—adult transition (also known as the vegetative phase change) and vegetative—reproductive transition represent the two main developmental transitions [10]. These transitions are important, firstly, plants can enter a reproductive stage under appropriate environmental conditions only during the adult phase [10]; therefore, the juvenile—adult phase change plays a critical role in plant development. In rice, this phase transition is usually associated with a series of changes in a range of species-specific traits, including the presence of a mid-rib, size and shape of the leaf blades, shoot apical meristem (SAM) size, and photosynthetic rate, etc [11]. Although the mechanisms underlying the vegetative phase change remain largely unknown, recent studies have revealed that miRNAs are involved in this vegetative phase change across species. Of them, miR156 and miR172 are well known for playing critical roles in the phase change of several species, including *Arabidopsis* [12,13], maize [14] and rice [15]. The expression of these two miRNAs is negatively correlated; thus, miR156 is intensively expressed during the juvenile phase to control shoot development, while miR172 is strongly expressed during the adult phase. miR156 targets the *SQUAMOSA PROMOTER BINDING PROTEIN LIKE (SPL)* transcriptional factors, which control the transition from juvenile to flowering stage by regulating the expression of a class of *MADS* box genes [13,16,17]. Over-expression of miR156 prolongs the juvenile-phase, produces more tillers, delays flowering, and reduces the number of spikelet [14,16,18,19]. On the other hand, miR172 targets *AP2*-like transcription factors, promoting both vegetative phase change and floral induction [20]. Over-expression of miR172 leads to earlier flowering and produces abnormal floral organs [21]. Together with the juvenile—adult transition, the vegetative—reproductive phase transition, known as the floral transition, is the most dramatic phase change in plant development. This transition is regulated by a complex genetic network that monitors the developmental state of the plant, as well as the environmental conditions such as photoperiod and level of phytohormones [16,22]. Recent molecular biological and genetic advances have revealed that *FLOWERING LOCUS T (FT)* in *Arabidopsis*, a long-day plant, and *Heading date 3a (Hd3a)* and *Rice FT-Like 1 (RFT1)* in rice, a short-day plant, encode florigen as a mobile leaf-derived signal aimed to trigger floral transition [23–25]. In addition, following the transition from the vegetative to the reproductive phases, the fate of the vegetative SAM changed, transforming into an inflorescence meristem (IM). Throughout this process, the stem cell must balance the maintenance of totipotent, undifferentiated stem cells and generation of differentiation cells [26]. In *Arabidopsis*, the WUSCHEL-CLAVATA (WUS-CLV) feedback loop pathway is one of the best-characterised signalling mechanisms involved in the regulation of meristem identity maintenance [27]. WUS is known to promote the expression of CLV3 and SAM maintenance, while CLV3 negatively regulates SAM maintenance by restricting the expression of WUS [27]. At the same time, recent studies in both maize and rice have suggested that the WUS-CLV feedback loop pathway is conserved in grasses [28–30].

microRNA171 (miR171) family is one of the most ancient and well conserved miRNA families known to date, which has been isolated from a large number of plant species, from mosses to flowering plants [31–33]. This family is known to target the *HAM* (*hairy meristem*) genes *(HAMs*, also known as *SCARECROW-LIKE6* or *LOST MERISTEMS*), which encode a member of the *GRAS* (*GAI-RGA-SCR*) plant-specific transcription factor family [34,35]. The *GRAS* genes play important roles in diverse cellular processes, including light and hormone signalling pathways and meristem maintenance [36]. Previous studies divided the *GRAS* family into 13 subfamilies based on phylogenetic data —*AtSHR*, *AtPAT1*, *AtSCR*, *AtSCL4/7*, *AtLAS*, *Os19*, *HAM*, *Os4*, *Pt20*, *DLT*, *AtSCl3*, *DELLA*, and *LISCL*, which have distinctly conserved domains and functions [37]. To date, the functions of some of the *GRAS* genes have been identified in rice. The DELLA protein OsSLR1 plays a role inhibiting gibberellin (GA) signalling [38]. *MOC1* encodes a GRAS protein that controls the formation of auxiliary meristems in rice [39]. *OsCIGR1* and *OsCIGR2* can be induced in the presence of the elicitor N-acetylchitooligosaccharide and exogenous gibberellins [40]. In *Arabidopsis*, miR171 highly expressed in the inflorescence, and it is known to regulate *HAM* expression through mRNA cleavage [41]. Over-expression of miR171 or loss of *AtHAM1*,*2*,*3* function produces pleiotropic phenotypes, including fewer cauline and rosette leaves, reduced shoot branching, increased chlorophyll content, shorter primary roots, and abnormal flower patterning [42,43]. In addition, *atham1*,*2*,*3* mutant exhibits loss of indeterminacy in both the shoots and roots, aberrant shoot phyllotaxis and lateral organs, and altered meristem morphology [43]. Further, a more detailed analysis of *atham1*,*2*,*3* mutant demonstrated that *HAM1* and *HAM2* are important to promote cell differentiation at the periphery of the shoot meristems and to help maintain their polar organization [44]. In barley, over-expression of miR171 has been also associated with pleiotropic phenotypes, including an extended vegetative phase, an increased number of short vegetative phytomers, and a delay in the differentiation of spikelet meristems into floral organs [45]. These results suggest that miR171 plays a conservative role in regulating meristem identity, but the regulation of the phase transition may be monocot-specific functions.

Set against this background, little is known about the function of miR171 and their targets in rice and how miR171 mediates the phase transition from vegetative to reproductive development. In this study, we identified a rice mutant resulting from T-DNA insertion at the promoter of *OsMIR171c* gene, where *OsMIR171c* was up-regulated. This mutant produced severely delayed-heading (flowering) phenotype. We found that the rice osa-miR171c-*OsHAMs* module is involved in the maintenance of shoot apical meristem indeterminacy and vegetative to reproductive phase change.

## Materials and Methods

### Plant materials and growth conditions

Rice (*Oryza sativa* L.) were grown in a controlled paddy of the South China Botanical Gardenduring natural growing seasons or in a greenhouse at 28°C for 14-h (day) and 10-h (night) circadian cycle in winter. This planting was permitted by South China Botanical Garden. The seedlings were grown in Hoagland’s Solution [46] under normal growth conditions, and samples were collected at different stages for expression pattern analyses. Rice *japonica* cultivar Zhonghua11 (ZH11, wild type) and mutant *dh* were used in this study.

### Quantitative RT-PCR analysis of gene expression

Small RNA and total RNA were extracted from different organs of rice using RNAiso for small RNA (Takara, Code No. 9753A) and RNAiso Plus for total RNA (Takara, Code No. 9108) and digested with DNase I (Takara, Code No. 2212) according to the manufacturer protocols. Reverse transcription for small RNA was performed using M-MLV Reverse Transcriptase (Promega, Cat#M1701) in a stem-loop RT-PCR reaction [47]. The specific reaction component and protocol were conducted as described previously [48]. Quantitative real-time RT-PCR (qRT-PCR) reactions for miRNA and mRNA were also performed as previously described [48]. The relative expression of the genes was normalized to the expression level of *U6* and *e-EF-1a* with biological repeats in triplicate. *U6* and *e-EF-1a* were used as internal controls for miRNA and mRNAs, respectively. All primers used are listed in S1 Table.

### RNA ligase-mediated 5′-rapid amplification of cDNA ends (5′-RLM-RACE)

To identify the cleavage sites of the target mRNAs, a modified 5′-RLM RACE was carried out following a protocol previously described [49]. Total RNA was directly ligated to a synthesised RNA adaptor (GCUGAUGGCGAUGAAUGAACACUGCGUUUGCUGGCUUUGAU¬GAAA), which has a 5′-hydroxyl group at both ends and thus can only ligate to 5′-phosphorylated RNA, including truncated products of miRNA-guided mRNA cleavage. The ligated product was directly reverse transcribed using an oligo-(dT) primer. The cDNA was amplified by nested PCR, and the final PCR product was gel purified and sub-cloned into the pGEM-T Easy Vector (Promega, Cat#A1360) for sequencing.

### Target prediction of miRNAs

The target genes of the miRNA were predicted using psRNA Target (http://bioinfo3.noble.org/psRNATarget/) with default parameters. The information on the target genes was obtained from Gene Bank and listed in S2 Table.

### Southern blot analysis

Southern blot analysis was performed following a previously described protocol (Roche, Cat#11745832910). For this analysis, ten μg of genomic DNA were digested using *Xho* I or *Xba* I. The probe was prepared from a PCR-amplified fragment of the hygromycin B phosphotransferase gene.

### Scanning electron microscopy (SEM)

Shoot apices were collected at different growth stages. Fresh samples were fixed for 24 h in 4% paraformaldehyde (Sigma, St. Louis, USA) and 2% glutaraldehyde (Sigma) in 0.1 M PBS (pH 7.2), and subsequently washed with PBS buffer (0.1 M, pH 7.2) and dehydrated through a graded alcohol series of 70, 85, 95, and 100% of ethanol, each for 30 min. To prepare them for SEM (JSM–6360LV, Hitachi, Tokyo, Japan) observation, the samples were then washed with 100% ethanol once, post-fixed in 1% (w/v) osmium tetroxide (Alfa Aesar, Massachusetts, USA) for 2 h, dehydrated in a freeze drier (JFD–310, Hitachi, Tokyo, Japan), and sputter-coated with gold palladium in 6 different 30-s bursts (JEE–420, Hitachi, Tokyo, Japan). The samples were analysed with a scanning electron microscope (S-3000N; Hitachi, Japan).

### Subcellular localization of OsHAMs

A green fluorescent protein (GFP) fusion protein was constructed using full-length *OsHAM* cDNA with a C-terminal fusion of the GFP clone under the control of a *CaMV35S* promoter. Rice protoplast preparation and transformation were conducted as previously described [50]. Subcellular distribution of the GFP fusion protein was examined using a confocal laser scanning microscope (ZEISS-510 Meta). Excitation was achieved using an argon laser at 488 nm (GFP), and emission of GFP was detected from 492 to 550 nm. Auto-fluorescence of chlorophyll was simultaneously detected between 650 and 730 nm. The images presented are average projections of 8–20 optical sections.

### Statistical analysis

Data were represented as mean ± standard deviation (SD). Student’s *t*-test was used to analyse all the data presented as the mean ± SE. A *p* value <0.01 or <0.05 was considered as statistically significant. The statistical analyses were performed in SPSS 12.0.

## Results

### Identification and molecular analyses of a late-heading rice mutant

**Fig 1:**
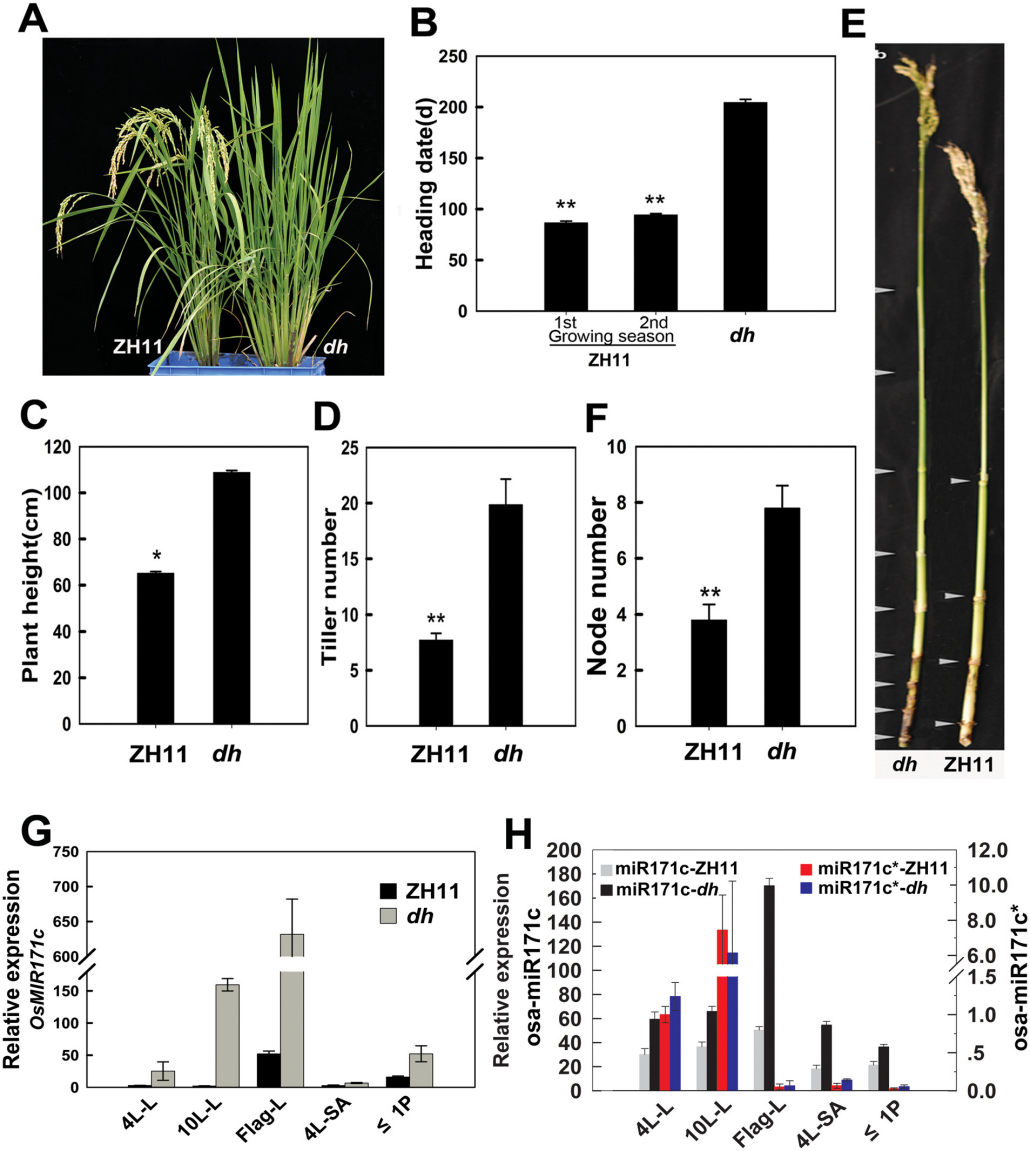
Main phenotypes of the delaying heading (*dh*) mutant and comparison of the osa-miR171c expression. (A) A wide-type plant Zhonghua 11 (ZH11) (left) and a *dh* mutant (right) under ND conditions after flowering of the wide-type plant. ZH11 was shown the plant from the 1^st^ growing season. (B) Statistical analysis of the growth time from sowing to heading of ZH11 at the 1^st^ and the 2^nd^ growing seasons compared with that of *dh* mutant. (C and D) Statistical comparison of plant height (C) and tiller number (D) between ZH11 and *dh* mutant. (E and F) *dh* mutant stems present more nodes than those from ZH11. (G and H) qRT-PCR expression comparison of *OsMIR171c* gene (G) and mature osa-miR171c and osa-miR171* (H) in different developing organs between ZH11 and *dh* mutant. Rice plants were grown in a paddy field in Guangzhou, China, during two normal growing seasons (1^st^ growing season from March to July, 2^nd^ growing season, from July to November), with normal fertilizer application. The data were presented from three-year experiments. Error bars indicate SD from at least 20 samples. *e-EF-1a* was used for *OsMiR171c* internal control and *U6* for osa-miR171c and osa-miR171* internal control. 4L-L and 10L-L, the 4^th^ leaf and the 10^th^ leaf; 4L-SA, shoot apex of 4-leaf stage seedlings; ≤1P, developing panicles with lengths ≤1 cm.

In our rice T-DNA insertion collection, one line displayed a serious delayed-heading (flowering) phenotype in the primary transgenic plant (T_0_). Heading date is one of the main agronomic traits in rice [51]. Thus, we aimed to characterise this trait because of its importance in agriculture. The late-heading phenotype was observed from the T_1_ to the T_3_ generations when the plants were grown in a paddy field under normal growth conditions (Fig 1A). For this mutant, the time required between sowing and heading was at least twice as long as the timing exhibited by the wild-type Zhonghua11 (ZH11) (Fig 1B). This mutant, therefore, was named as *dh (delayed heading)* mutant. Because *dh* mutant continues growing and tillering during the long vegetative stage, it eventually produces more tillers (Fig 1D), higher plants (Fig 1C), and more nodes (Fig 1E and 1F) than that by the wild-type ZH11.

We performed molecular analyses to characterize the *dh* mutant (S1 and S2 Figs). Southern blot analysis showed that the *dh* mutant presented one T-DNA insertion locus (S1B Fig). Thermal asymmetric interlaced (Tail) PCR [52] was used to identify the T-DNA insertion site. The sequence of the genomic fragment flanking the T-DNA insertion site showed that the T-DNA was inserted in the promoter region of the gene Os04g0623901; therefore, the gene is downstream of *Ubiquitin* and the *35S* promoter in the *dh* mutant, and there is a double-enhancer in the *35S* promoter (S1A Fig), which has observed being able to activate the downstream gene [53]. The corresponding mRNA of Os04g0623901 (*OsMIR171c*) is AK242153, which is predicted to encode a precursor of the osa-miR171c. The results obtained by qRT-PCR showed that the expression of *OsMIR171c* was up-regulated at least seventy-fold in the leaves of *dh* mutant (Fig 1G and S1C Fig), compared with that of the ZH11. The late-heading phenotype of the *dh* mutant was observed in the T_0_ transgenic rice, suggesting that the mutation may be dominant. To verify this, we performed a co-segregation analysis between the late heading and T-DNA insertion heterozygous plants (S2 Fig). The late-heading phenotype co-segregated with T-DNA, and the segregation ratio obtained for heterozygous plants in a three generation family was well within the expected 3:1 ratio (χ^2^, *p* = 0.05; S3 Table). These data suggested that *dh* mutant present a single T-DNA insertion site, and the late-heading phenotype is caused by a single sporophytically controlled Mendelian locus. Similarly, over-expression of miR171 has been also associated with an extended vegetative phase in barley [45]. Therefore, we deduced that T-DNA insertion enhances *OsMIR171c* expression, which consequently leads to the appearance of the late-heading phenotype in *dh* mutant.

### Mature osa-miR171c is the main up-regulated product in the *dh* mutant

To understand the biological function of osa-miR171c, we first searched the miRNA database (miRBase) [54], and found that eleven osa-miR171 have been deposited in the database. Sequence alignment indicated that miR171c is conserved across a variety of plant species, and that there are fourteen identical conserved nucleotide positions in two blocks of conserved regions (S3A Fig). Of them, seven *OsMIR171* genes generate one identical type of mature osa-miR171 (*b/c/d/e/f/m/n*). The *OsMIR171c* gene is transcribed as a long transcript, the osa-miR171c primary transcript (AK242153), which was confirmed by our RT-PCR sequencing result (S3B Fig).

The osa-miR171c precursor can be processed into two different mature forms, namely, osa-miR171c and osa-miR171c*; therefore, we examined the expression level of the *OsMIR171c* transcript (Fig 1G) and the two mature osa-miR171c forms (Fig 1H) in the *dh* mutant by qRT-PCR. The expression level of mature osa-miR171c was up-regulated by 0.5- to 3-fold in different developing organs, compared to that of ZH11; while the level of mature osa-miR171c* did not differ between the ZH11 and the *dh* mutant (Fig 1H). In addition, the expression level of osa-miR171c was generally much higher than that of osa-miR171c* in all the tissues examined. However, the elevated levels of mature osa-miR171c (0.5- to 3-fold) were not directly related to the up-regulated level of *OsMIR171c* (> 70 fold; Fig 1G and 1H and S1C Fig), it might be explained by that the mature osa-miR171c also is the product of the *OsMIR171b/d/e/f/m/n* genes (S3A Fig). These results suggested that mature osa-miR171c is the predominant form between the two mature osa-miR171c forms, and it is clearly up-regulated in the *dh* mutant.

### Four *OsHAMs* transcription factors are the targets of osa-miR171c in rice

**Fig 2:**
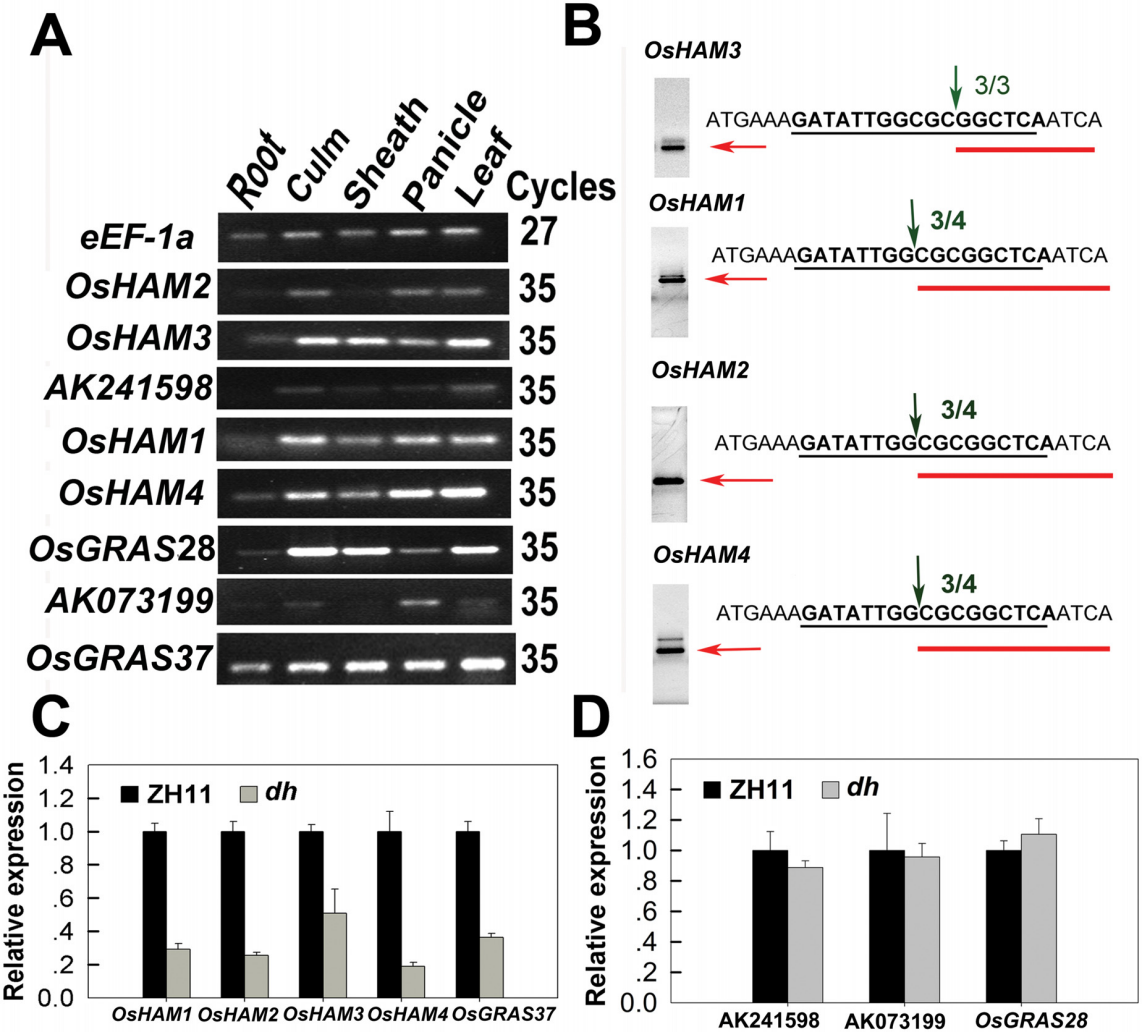
Four *OsHAM* transcription factors are targets of osa-miR171c. (A) Organ-expression profiles of the eight predicted targets of osa-miR171c in ZH11 at the heading stage by semi-RT-PCR. (B) Identification of cleavage sites of the target mRNAs in ZH11 using 5′-RLM-RACE. miRNA binding sites are underlined in black and the 5′ end of the cleaved mRNA in red. The numbers in green refer to the ratio of 5′-RACE clones and the arrows indicate the cleavage sites. The agarose gel images on the left show the bands corresponding to the amplified 3′ cleavage products. (C and D) qRT-PCR expression analysis shows down-regulated (C) and unchanged (D) target genes of osa-miR171c in *dh* mutant leaves. *e-EF-1a* was used as internal control in A, C, and D.

The psRNA target server (http://plantgrn.noble.org/psRNATarget) predicted the existence of nine genes (S2 Table) as the targets of osa-miR171c. Eight of them were predicted to be cleaved under osa-miR171c direction, except for AK101142, which may be translationally repressed by osa-miR171c. Therefore, for another eight putative targets except for AK101142, we could check the expression changes in the WT and *dh* mutant by qRT-PCR. Semi-qRT-PCR analysis showed that these eight genes were expressed in the leaves of ZH11 (Fig 2A); subsequently, rice leaves were used to detect the mRNA expression level of the eight putative targets by using qRT-PCR with primer pairs spanning the miR171 cleavage site. The expression of five genes (Os02g0662700, Os02g0663100, Os04g0555000, Os06g01052000, and Os10g0551200; Fig 2C) were down-regulated in the leaves of the *dh* mutant; however, the expression of the other three genes (Fig 2D) showed no obvious change, when compared with those of ZH11.

To further determine whether the 5 target genes are cleaved in the osa-miR171c direction, a 5′-RLM-RACE assay was performed using RNA isolated from mixed samples of the shoots and the panicles of ZH11 (Fig 2B). Sequencing of the 5′-RLM-RACE clones indicated that the cleavage sites of four genes (Os04g0555000, Os02g0662700, Os02g0663100, and Os06g0105200) were within the osa-miR171c-*HAM* complementary region (Fig 2B). The cleavage site of *OsHAM3* was different from the others. No cleavage was detected for Os10g0551200 in any of the tested samples. Taken together, we concluded that at least four *OsHAM* genes could be the targets of osa-miR171c in rice.

The four targets belong to the *HAM* subfamily of the extensive *GRAS* family of plant-specific transcription factors (S4 Fig), and they contain all the highly conserved domains of the *GRAS* family, except for the RVER domain in their C-termini SAW motif (S4A Fig) [35]. A phylogenetic analysis based on HAMs amino acid sequences from HAMII (containing the miR171-binding sequence) of 36 representative species showed that the HAMs formed two distinct groups of monocot and eudicot [43] (S4B Fig). The four osa-miR171c targets were all grouped in the monocot clade; we named these four genes as *OsHAM1* for Os02g0662700, *OsHAM2* for Os02g0663100, *OsHAM3* for Os04g0555000 and *OsHAM4* for Os06g0105200. OsHAM4 appeared to be evolutionary distant from the other OsHAMs (S4B Fig); thus, we deduced that OsHAM4 may have another function. Three OsHAM proteins were predicted to have a nuclear Localization signal except for OsHAM4 (S5A Fig). To investigate whether the four OsHAM proteins are transported to the nucleus as other transcription factors, they were transiently expressed in rice protoplasts (S5B Fig). Three of the four OsHAM-GFP fusion proteins were localized predominately in the nucleus except for the OsHAM4-GFP fusion protein, which demonstrated that OsHAM1/2/3 proteins could be transported to the nucleus. OsHAM4 may require the interaction of other proteins to target the nucleus.

### osa-miR171c controls the expression change of four *OsHAMs* in shoot apex during floral transition

**Fig 3:**
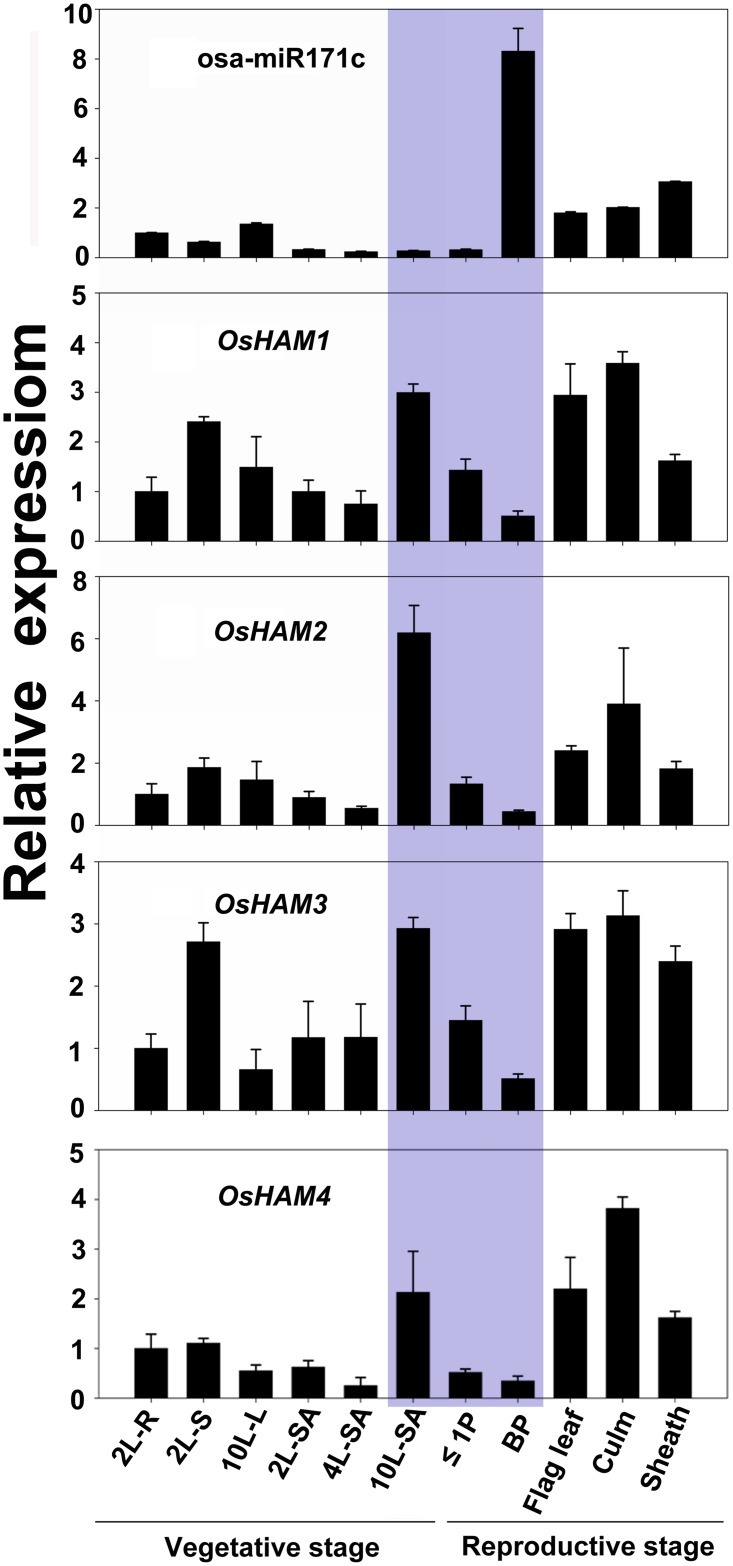
Spatial and temporal parallel expression analyses of osa-miR171c and *OsHAMs* in different ZH11 organs at vegetative and reproductive stages. Data were calculated from three replicates. ZH11 plants were grown in a paddy for microRNA and RNA extraction. The shadow showed inverse correlation of expression change between osa-miR171c and 4 *OsHAM* genes during transit from vegetative to reproductive stage. *e-EF-1a* and *U6* were used as *OsHAMs* and osa-miR171c internal controls, respectively. Expression level of osa-miR171c and 4 *OsHAMs* was normalized with their expression at 2L-R stage, respectively. 2L-R, roots of 2-leaf stage seedlings; 2L-S, 2-leaf stage seedlings; 10L-L, the 10th leaf; 2L-SA, shoot apex of 2-leaf stage seedling;4L-SA, shoot apex of 4-leaf stage seedling;10L-SA, shoot apex of 10-leaf stage seedling;≤1P, developing panicles with a length of ≤1 cm; BP, booting panicle.

To investigate how osa-miR171c controls *OsHAM* expression in developing organs in rice, we analysed the parallel expression of osa-miR171c and its targets in ZH11 using qRT-PCR (Fig 3). First, we tested the expression pattern of osa-miR171c in developing organs using stem-loop qRT-PCR (Fig 3,). The results showed that osa-miR171c was expressed in all the tested organs. The highest expression levels were detected in the booting panicle and the weakest expression levels were in the shoot apex of different developmental stages. In general, osa-miR171c was mainly expressed in reproductive organs, which was consistent with a role regulating the timing of floret initiation and development in rice and consistent with previous data published for *Arabidopsis* and barley [45,55].

Second, primer pairs spanning the cleavage sites of the target mRNAs were used in a qRT-PCR reaction to detect intact transcripts of the target genes (Fig 3). The four *OsHAM* genes shared a similar expression pattern. They were expressed in all organs, but their transcript abundance varies considerably among them. The highest expression of the four genes was detected in the shoot apex of the 10-leaf stage seedlings at the onset of floret transition. On the other hand, the lowest expression levels were detected in the booting panicle. In the shoot apex, the expression of the four *OsHAM* gradually increased with shoot development. When SAM transform into the floral meristem, the expression levels decrease sharply. These expression profiles suggested that *OsHAMs* plays a role in controlling phase transition.

In conclusion, osa-miR171c expression level was very low at the 10-leaf stage, when plants were during the late adult vegetative phase and immediately prior to flowering initiation, especially in the shoot apex; however, expression levels increased steadily during panicle development (Fig 3). In contrast, *OsHAMs* were expressed intensively during the adult vegetative phase, although their expression gradually declined along with vegetative to reproductive phase transition (Fig 3). These data strongly support the hypothesis that the gradual decrease of *OsHAM* expression in the shoot apex during phase transition is inversely proportional to osa-miR171c expression level. However, in other organs, osa-miR171c level was not always inversely correlated with the expression of the four targets, indicating that osa-miR171c and its target genes interact in a complex manner in rice.

### Light regulates the expression of osa-miR171c and the four *OsHAMs*

**Fig 4:**
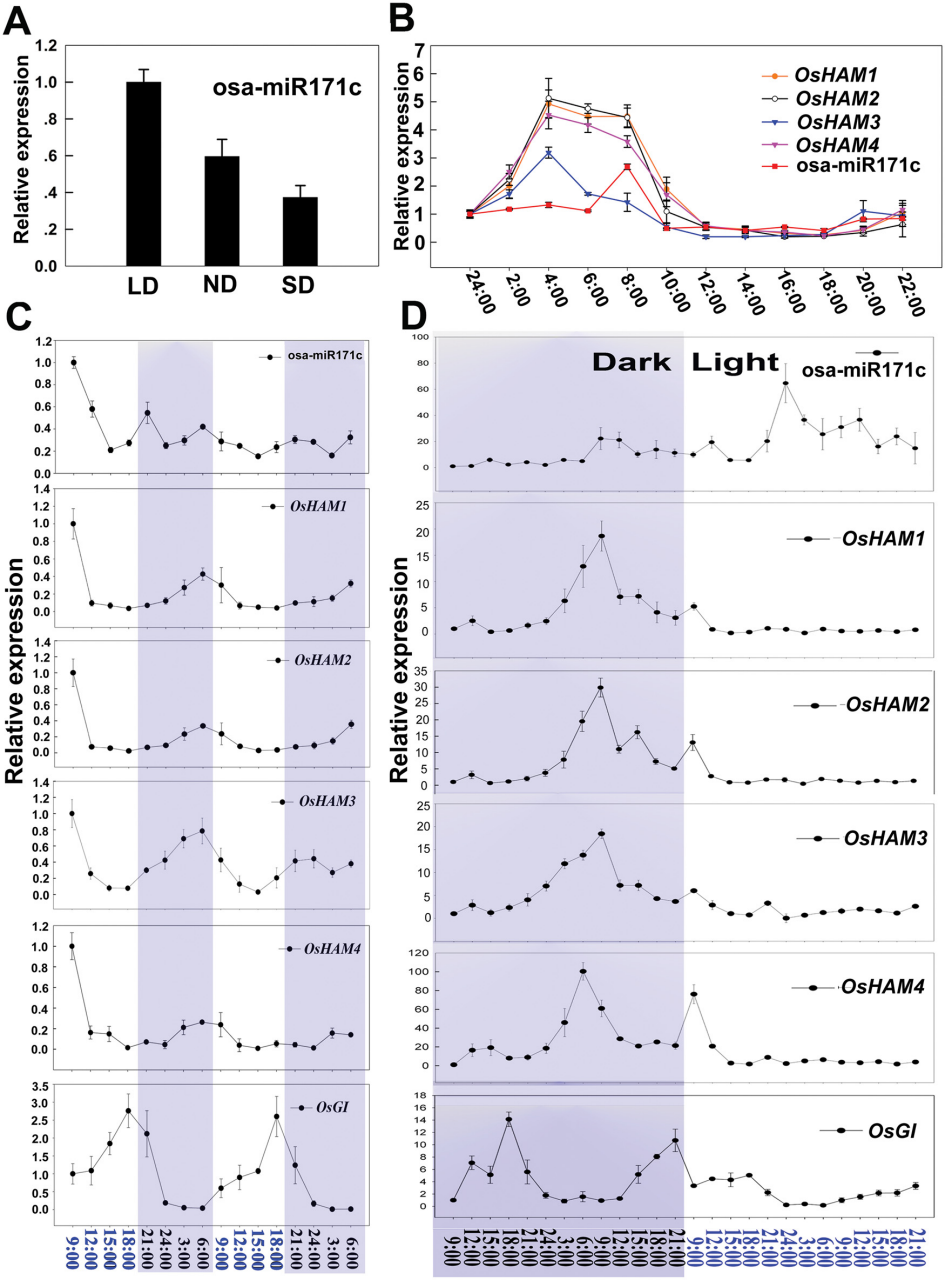
Light regulates expression of osa-miR171c and *OsHAMs*. (A) Expression level of osa-miR171c under nature day (ND), long day (LD, 14-h light), and short day (SD, 9-h light) conditions. (B) Transcript levels of osa-miR171c and *OsHAMs* changed during through the 24-h day/night period. (C) Transcript levels of osa-miR171c and *OsHAMs* changed during the day/night period (48 h). (D) Expression of osa-miR171c and *OsHAMs* in continued dark and continued light conditions. *e-EF-1a* and U6 were used for *OsHAMs* and osa-miR171c internal controls, respectively.

Nine *cis*-elements in *OsMIR171c* promoter were predicted to be involved in the plant response to light conditions (S4 Table). For this reason, we investigated the relation between osa-miR171c and *OsHAMs* expression under different photoperiodic conditions (Fig 4). During a day and night cycle, osa-miR171c expression increased rapidly with sunlight and reached a peak in the early morning (6:00 to 10:00), it then decreased rapidly and was kept at a relative low levels throughout the day (Fig 4B). In addition, osa-miR171c expression level was higher under long days than under short day conditions (Fig 4A). In contrast, the expression of the four *OsHAMs* increased during the evening to reach a peak at midnight and then decreased at dawn (Fig 4B). The expression levels of osa-miR171c were inverse correlated with those of *OsHAMs* following a natural day/night photoperiod.

The expression data obtained using RiceXpro (http://ricexpro.dna.affrc.go.jp/) showed that the expression of the four *OsHAMs* is diurnal changed (data not shown); therefore, we analysed the circadian pattern in the expression of osa-miR171c and *OsHAMs*. Under natural photoperiodic conditions, osa-miR171c expression showed a weak diurnal pattern with the expression level peaking just immediately after dawn (Fig 4C). On the other hand, *OsHAMs* expression exhibited an obvious diurnal expression pattern with the expression levels peaking at night (Fig 4C). To distinguish whether the expression of osa-miR171c and *OsHAMs* was mediated by a circadian rhythm control or only by light, we analysed their expression under continuous light and under continuous darkness, by using the rice diurnal gene *OsGI* as a control [56]. The expression of osa-miR171c was reduced to a relative low level under continuous darkness; however, such reduction was not observed when the plants were grown under continuous light (Fig 4D). Similarly, the expression of *OsHAMs* was inhibited under continuous light while during continues darkness it exhibited the similar diurnal pattern than that observed under natural light conditions (Fig 4D). These results suggested that osa-miR171c and *OsHAM* expression was primarily regulated by light, rather than indirectly through changes in the circadian rhythm.

### Up-regulation of osa-miR171c drastically delays rice heading date

**Fig 5:**
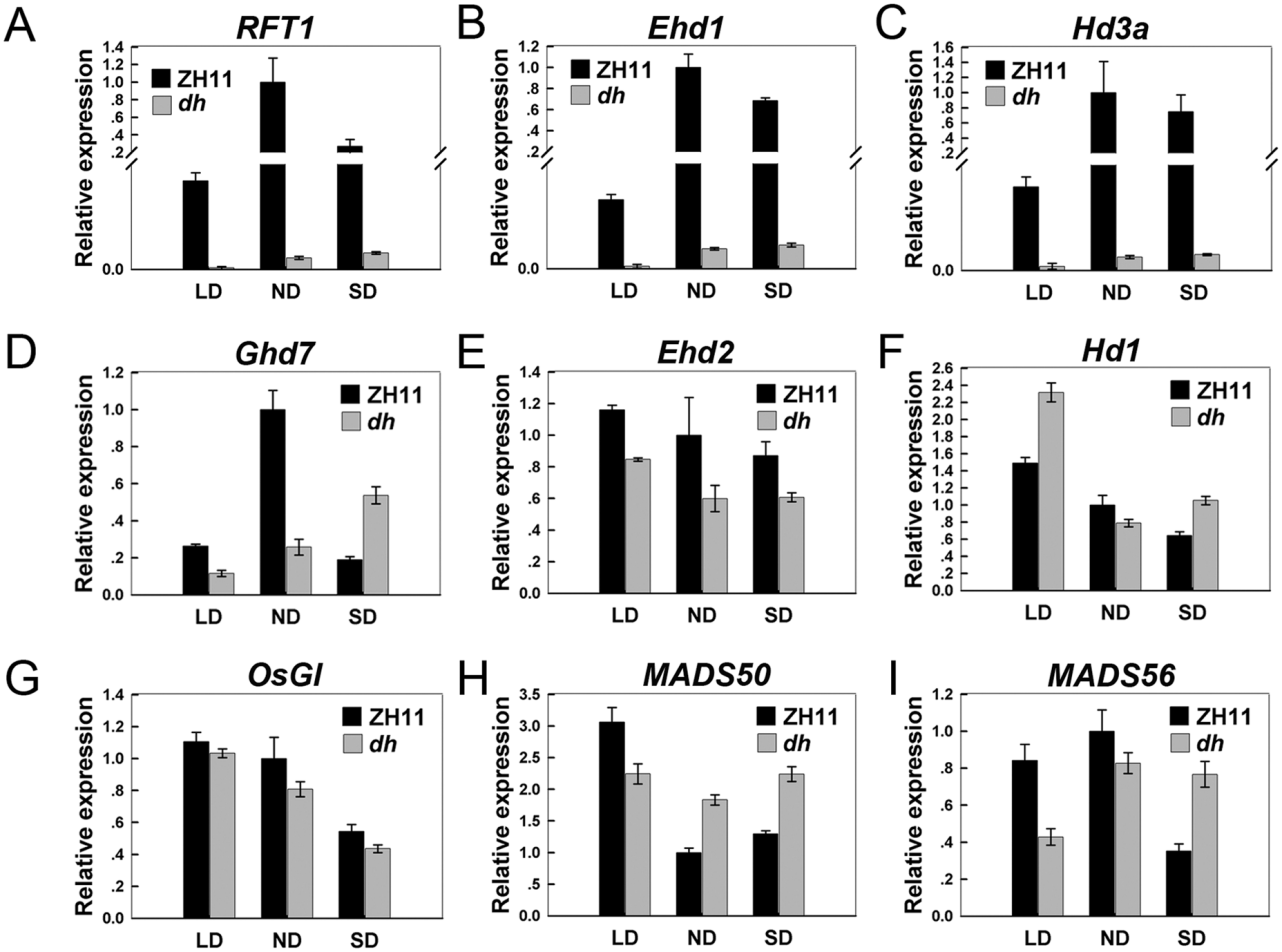
Expression comparison of nine flowering-related genes in the rice flowering regulation model [57], between ZH11 and *dh* mutant under SD, ND and LD conditions. Total RNAs were extracted from leaves, collected from 21-day-old plants grown under short days (SD, 9-h-day/15-h-night), from 21-day-old plants grown in a field under natural day conditions (ND) and from 30-day-old plants grown under long days (LD, 14-h-day/10-h-night). Values are shown as means of two biological replicates. Error bars indicate standard deviation. *e-EF-1a* was used as internal control.

The most conspicuous feature of the *dh* mutant was the occurrence of delayed heading date (Fig 1A). To test whether the late-flowering phenotype of the *dh* mutant can be changed under different photoperiods, we grew the *dh* mutant under different photoperiodic conditions. The results showed that the late-flowering phenotype of the *dh* mutant did not change with the photoperiodic conditions (data not shown). We subsequently compared the expression of nine flowering-related genes of the rice flowering regulation model [57] between the *dh* mutant and ZH11 using qRT-PCR (Fig 5). Leaf samples were collected during the floral transition stage following direction [58], from 21-days-old plants (growing under short and natural day conditions) and 30-days-old plants (growing under long day conditions). The leaf samples were collected 2 h after dawn, when the transcription of these genes was at the highest level [58]. Among the 12 genes investigated, 3 genes (*Hda3*, *RFT1*, and *Ehd1*) showed significantly lower transcription levels in the *dh* mutant than in the ZH11 for the three photoperiodic conditions studied (Fig 5A–5C), while the other six genes (*OsHd1*, *OsEhd2*, *OsGhd7*, *OsMADS56*, *OsG1*, and *OsMADS50*) did not show a significant difference in expression between *dh* mutant and ZH11 (Fig 5D–5I). These results also suggested that osa-miR171c and its targets may regulate a flowering pathway different from the pathway that studied before.

**Fig 6:**
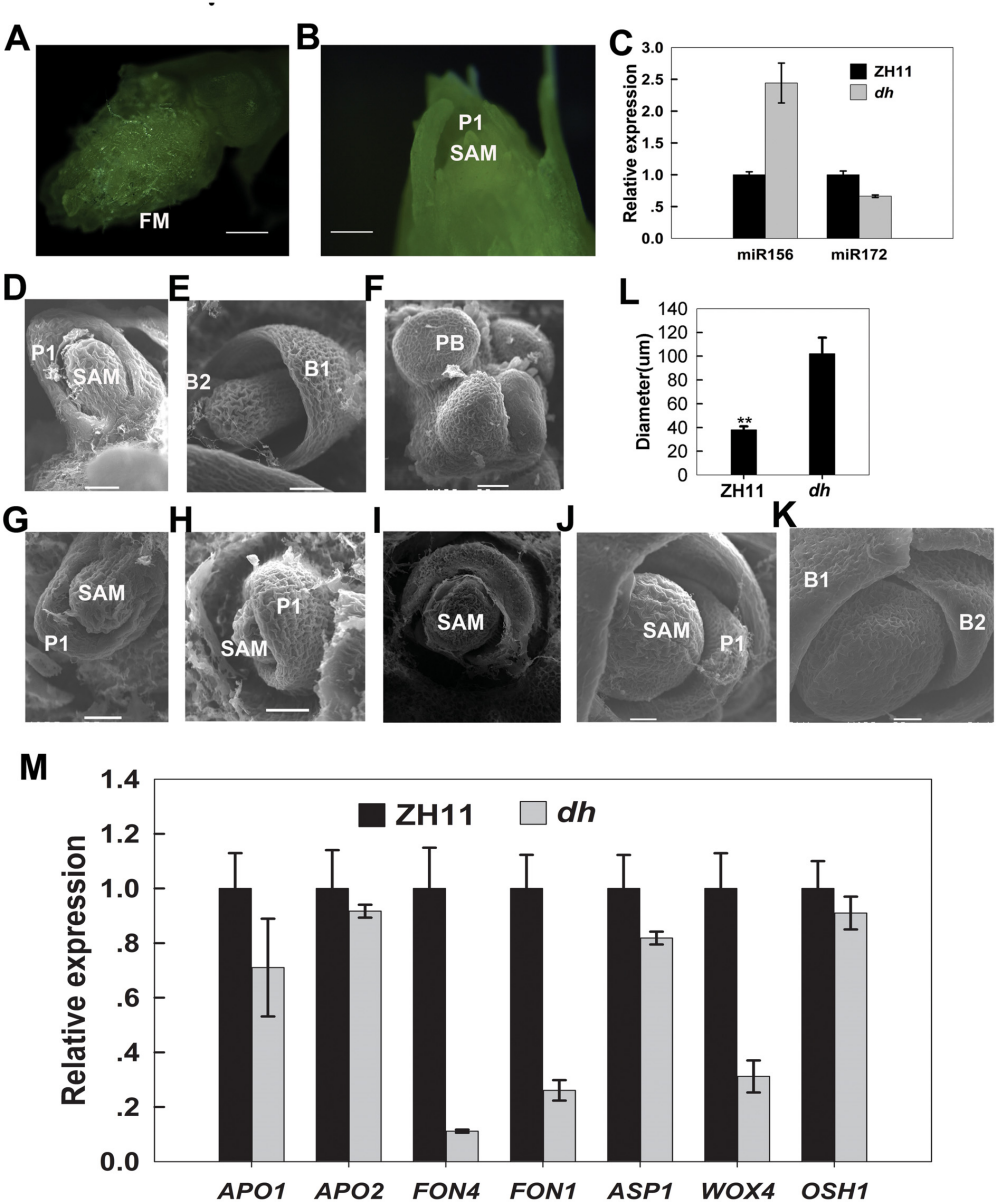
Prolonged development of shoot apical meristem (SAM) in *dh* mutant. (A and B) Shoot apex of ZH11 (A) and a *dh* mutant (B) at 40 days after germination (DAG). (C) Expression levels of miR156 and miR172 in the fifth leaf of ZH11 and *dh* mutant. (D-E and G-H) SAM morphology of ZH11 (D, E) and a *dh* mutant (G, H) at the 4 leaf-stage and the 10 leaf-stage. (F and I) Shoot apex of ZH11 (F) and a *dh* mutant (I) at 30 DAG. (J and K) Shoot apex of a *dh* mutant at 80 DAG (J) and a *dh* mutant at 100 DAG (K). (L) Statistical analysis of SAM diameters of ZH11 and *dh* mutant before flowering transition. (M) Expression analysis of SAM identity genes, and floral identity genes in the shoot apex of ZH11 and *dh* mutant. *e-EF-1a* was used as internal control. FM, floral meristem; P1, leaf primordial; SAM, shoot apical meristem; B1, first bract; B2, second bract; PB, primary branch. Scale bars = 20 μm. ** represents significant difference compared to ZH11 (*p* < 0.01). Error bars indicate SD from at least six measurements.

We further observed the morphological change of shoot apical meristem (SAM) in *dh* mutant (Fig 6). SAM of ZH11 stopped producing leaves and converted into an inflorescence meristem (IM) (Fig 6A), with the initial SAM elongation observed at 40 days after germination (DAG); however, the *dh* mutant continued to produce new leaves at this stage (Fig 6B), indicating that the juvenile—adult transition was delayed in *dh* mutant. SAM in *dh* mutant converted into IM after 150 DAG, at a stage when the ZH11 seeds had been already harvested for a long time. To further understand the mechanism underlying the delay in the juvenile—adult phase transition, we used qRT-PCR to examine osa-miR156 and osa-miR172 expression (Fig 6C). In leaves of *dh* mutant 5-leaf stage seedlings, osa-miR156 expression level was higher than that in ZH11; however, osa-miR172 expression did not differ between them. miR156 has been demonstrated to be related with a delay in the juvenile-adult phase change [19]. Therefore, we deduced that osa-miR171c and its targets played a role in the juvenile-adult phase change.

Finally, scanning electron microscope (SEM) was used to observe SAM morphological changes during vegetative to reproductive phase transition. SAM did not differ morphologically between *dh* mutant and ZH11 during the seedling stage (Fig 6D and 6G). However, when SAM of ZH11 underwent transition from vegetative to reproductive phase to generate rachis branches (Fig 6E and 6F), SAM in *dh* mutant still maintained the vegetative identity and continued to produce more leaves than those in ZH11 (Fig 6H and 6I). At the same time, SAM enlarged gradually during development in *dh* mutant. At 80 DAG, SAM showed a flat shape in *dh* mutant (Fig 6J), and at 100 DAG, it became even bigger and flatter (Fig 6K), reaching a three times bigger size than of those in the ZH11 (Fig 6L).

To understand the causes leading to the delayed heading observed in *dh* mutant, we compared the transcription pattern of four SAM identity genes expressed in shoot apex (before the period which convert to the IM) by qRT-PCR between *dh* mutant and ZH11 (Fig 6M). The results indicated that the genes *OsFON1*, *OsFON4* (orthologs of *Arabidopsis CVL1* and *CLV3*, respectively), and *WOX4* were significantly down-regulated in the *dh* mutant, whereas *OSH1* remained unchanged. As a result, similar to the function of miR171 in *Arabidopsis*, osa-miR171c affects the maintenance of SAM indeterminacy by regulating WUS-CLV feedback loop. Therefore, we deduced that osa-miR171c and its targets can delay the heading date by influencing the maintenance of SAM identity.

### Up-regulation of osa-miR171c results in abnormal reproductive organs

**Fig 7:**
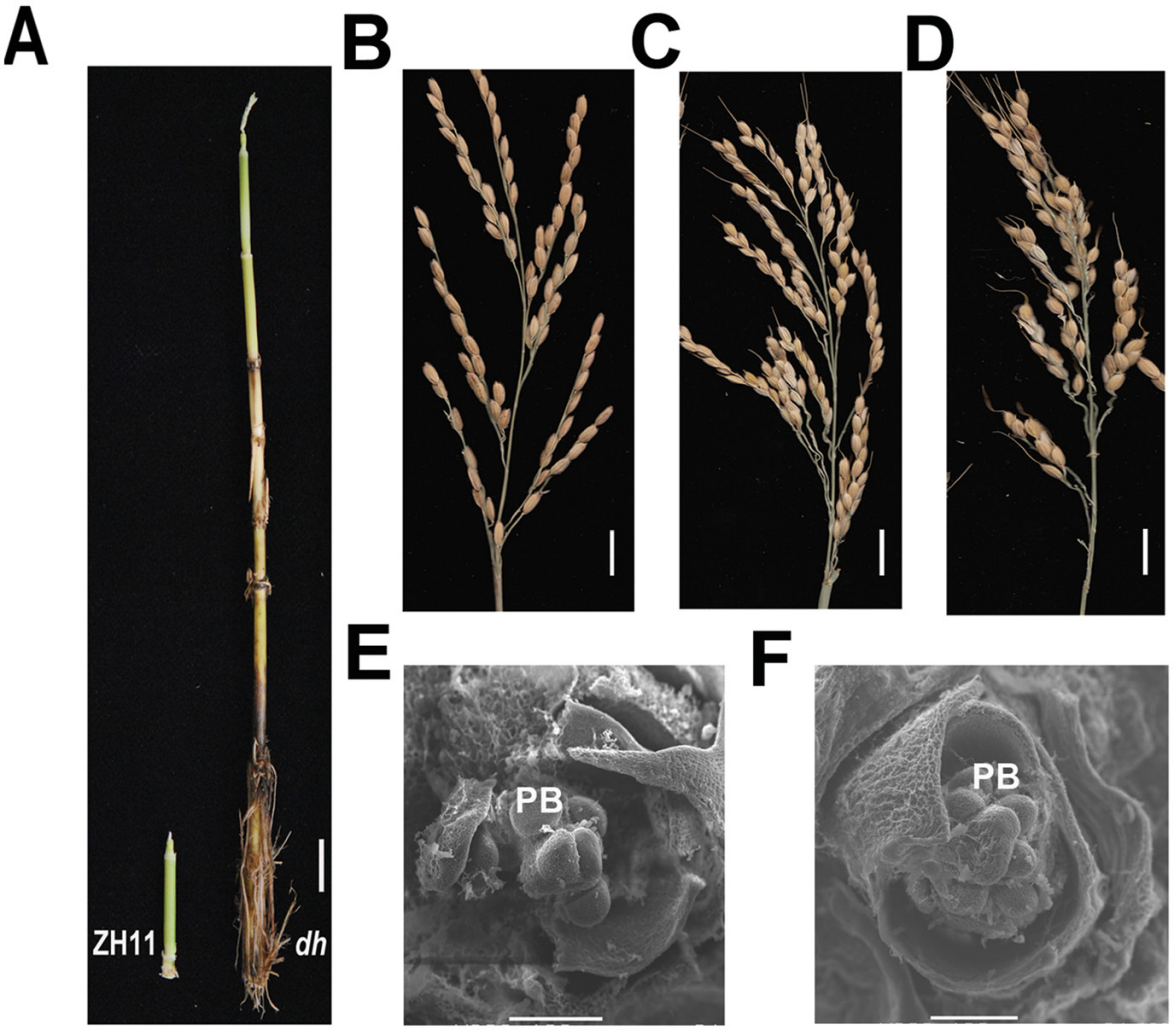
Reproductive organ abnormalities in *dh* mutant. (A) Phenotype of the culms with young panicles. (B—D) Panicle structure of ZH11 (B) and a *dh* mutant (C, D). (E and F) SEM images showing the formation of primary branches in a ZH11 (E) and a *dh* mutant (F). PB, primary branch; Bar = 1cm (A—D), 100 μm (D, E).

We also analysed the effect of osa-miR171c in the development of reproductive organs (Fig 7). The results showed the diameter of *dh* mutant stems was significantly wider than that of ZH11 (S6 Fig) and the nodes number of *dh* mutant were more (Fig 1E and 1F). The panicle of *dh* mutant showed pleiotropic morphological abnormalities, such as shorter and more primary branches, dense spikelet and longer awn (Fig 7C and 7D). The number of primary branches increased almost two-fold from 7 ± 1 in ZH11 (Fig 7B) to 12 ± 2 in *dh* mutant (Fig 7C and 7D). In *dh* mutant, the spikelet agglomerated, forming an extremely condensed pattern in the majority of the primary branches; on the other hand, at the top of the lemma in the mutant, a long awn was observed (Fig 7C and 7D). SEM of inflorescence and spikelet development showed that formation of the primary branches, secondary branches, and spikelet did not differ between ZH11 and *dh* mutant (Fig 7D and 7E).

The transcription of genes (*APO1*, *APO2*, and *ASP1*) related to primary branch development [59,60], were compared by qRT-PCR (Fig 6M) in shoot apex undergoing transition to inflorescence meristem between *dh* mutant and ZH11. The results showed that the expression of *APO1*, *APO2*, and *ASP1* did not differ between ZH11 and *dh* mutant. However, SAM in *dh* mutant was very large and produced a larger inflorescence meristem than ZH11 (Figs 6E, 6K, 7E and 7F). In addition, it produced more primary branches, which agrees with a previous report showing that the primary branch number is determined by the initial size of the reproductive apex [61]. These results suggested that osa-miR171c and its target genes are involved in different aspects of meristem function, including initiation and maintenance.

### Up-regulation of osa-miR171c leads to abnormalities in leaf morphology

To investigate whether osa-miR171c affects the development of vegetative organs, we analysed leaf morphology using SEM (S8 Fig). Rice leaves typically show a parallel venation pattern, and the three major longitudinal veins—mid vein (MV), large vein (LV), and small vein (SV) usually lie parallel along the distal axis of the leaf [62,63]. The number of SVs between LVs was quite irregular in leaf blades of *dh* mutant. ZH11 leaves showed five SVs (S8A Fig); however, the SV number ranged from three to six in *dh* mutant (S8B Fig). The diameter of the mastoideus was larger in *dh* mutant (S8D Fig) than in ZH11 (S8C, S8E Fig) on the adaxial side of the leaves. These data showed that osa-miR171c also affects the development of vegetative organs.

## Discussion

**Fig 8:**
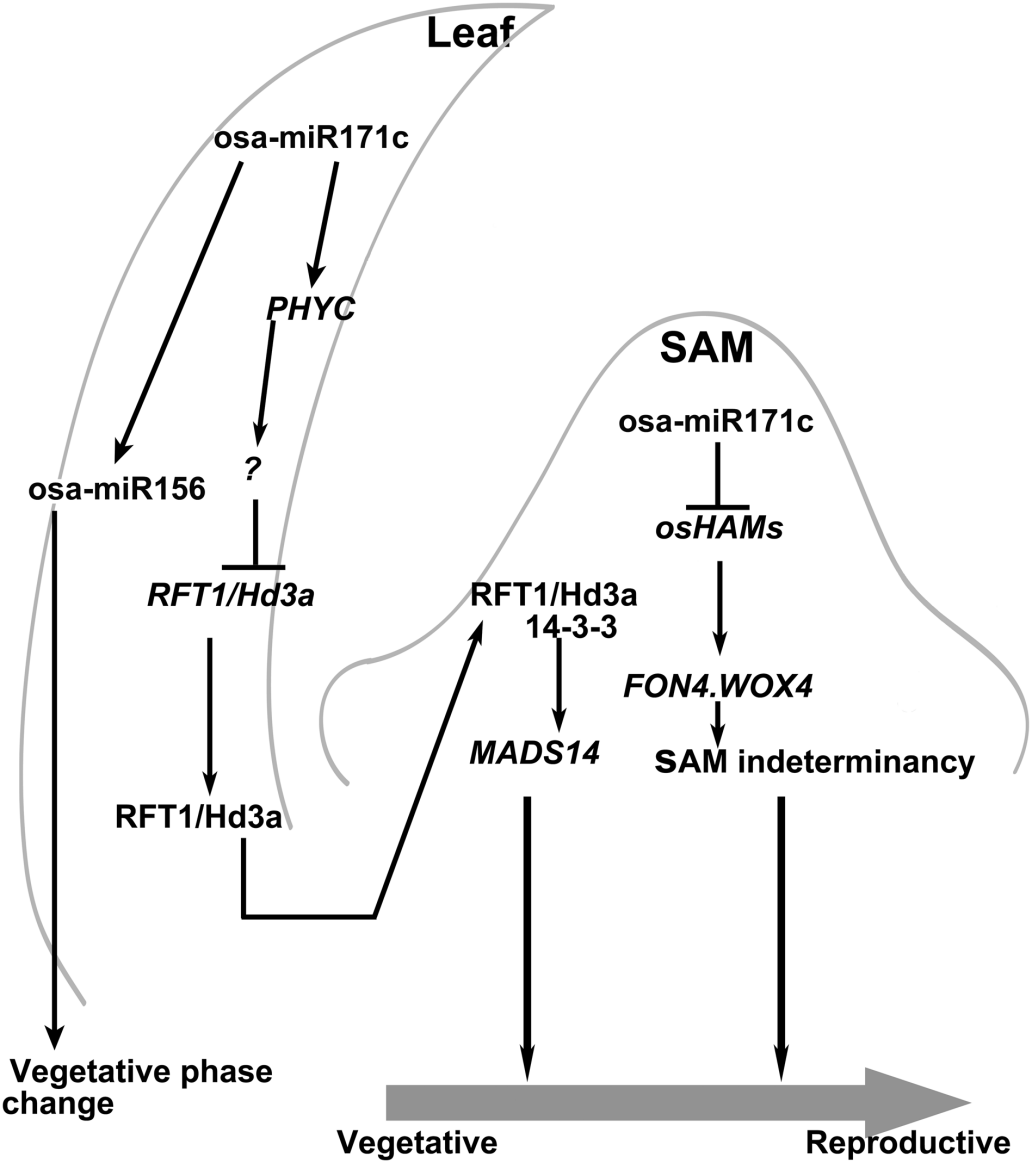
A model of *osa-miR171c* in the phase change pathway. In the leaf, miR171 delays juvenile—adult phase change mainly by regulating miR156. In the late adult phase, osa-miR171c could affect the expression of florigens *RFT1* and *Hd3a* by altering the expression of *OsPHYC*. *RFT1* and *Hd3a* proteins move to the SAM, where FD and 14-3-3 is expressed. The module is proposed to activate the transcription of downstream floral promoter genes such as MADS14 and MADS15. At the same time, osa-miR171c and its targets affect SAM maintenance by regulating the expression of *FON4*, *WOX4* genes. Potential influences are indicated by plain arrows (positive associations) and plain T-ended lines (negative associations).

The miR171 family are highly conserved across land plants, from moss to flowering plants of both monocots and dicots [33]. Based on the characterization of miR171 over-expressing transgenic plants, the miR171 family has been shown to be not only involved in the negative regulation of shoot branching in *Arabidopsis* [42], but also in the mediation of phase transitions and floral meristem determinacy in barley [45]. Here, we identified that four *OsHAMs* transcripts are cleaved under direction of osa-miR171c in rice (Fig 2B and 2C), and the up-regulation of osa-miR171c resulted in prolonged vegetative phase and serious delayed rice heading date. We found that the osa-miR171c*-OsHAMs* module may be involved in three different pathways controlling phase transition (Fig 8).

### Interaction between osa-miR171c and *OsHAMs*

*HAM* family has been predicted to be the target of miR171 in *Arabidopsis*, *Nicotiana benthamiana*, barley, and *Larix kaempferi (Lamb*.*) Carr* [41,42,45,64]. In rice, there are seven putative *HAM* genes [37], five of them contain osa-miR171c recognition sites (S2 Table), while the other two *HAMs* genes are the putative rice homologs of *NSP2* [65]. In our study, we showed that osa-miR171c can cleave *OsHAM1*, *OsHAM2*, *OsHAM3*, and *OsHAM4* mRNAs (Fig 2B and 2C). Several lines of evidence support this conclusion: first, the over-expression of osa-miR171c resulted in a reduction in *OsHAMs* transcripts; second, 5′-RLM-RACE data showed that *OsHAMs* were the target of osa-miR171c; and third, the expression of *OsHAMs* was inversely correlated with osa-miR171c expression in shoot apex during floret transition. These results showed that at least four *OsHAMs* could be the target genes of osa-miR171c in rice.

Positive and negative feedback loops of miRNA-target modules have been described for the miRNA regulation in plants [13]. In *Arabidopsis*, miR171a, miR172b, and miR156a are found to be positively regulated by their target genes [13,66]. We found that the inverse expression correlation between four *OsHAMs* and osa-miR171c was mainly observed in the shoot apex during floret transition (Fig 3). In other organs, the expression patterns of osa-miR171c and *OsHAMs* nearly coincided in the temporal scale (Fig 3), similar to that reported in barley and *Arabidopsis* [42,45]. Therefore, we proposed that this expression pattern is related to the existence of a regulatory feedback loop between *HAMs* and *MIR171* genes.

### The osa-miR171c*-OsHAMs* module negatively regulates phase change in rice

Our findings suggested that osa-miR171c represses the juvenile—adult phase change in rice by regulating the expression of miR156, in addition, up-regulation of osa-miR171c caused the delayed vegetative phase transition traits, such as continuously produce of the leaf primordium (Fig 6B). miR156 and miR172 have been shown to play a critical role in the vegetative phase change in several plant species [13,18]. The maize *Cg1* mutant has been shown to extend juvenile phase through the over-expression of miR156 [14]. Down-regulation of the target gene *Glossy15* of miR172 exhibited a shortened juvenile phase [67]. In rice, the phenotype associated with the over-expression of miR156 is similar to that of *dh* mutant, delaying the heading date and increasing tiller numbers [68,69]. A phenotype over-expressing miR172 was not observed in *dh* mutant [21]. In the *dh* mutant, miR156 expression was up-regulated and miR172 expression remained unchanged (Fig 6C), which is similar to the expression patterns of miR156 and miR172 found in the miR171 over-expressing barley [45]. Recently, observations by Xue et al. [66] showed that the targets of miR156 and miR171 *HAM-SPL* can interact with each other to affect a series of developmental events, including flowering, by repressing SPL activity in *Arabidopsis*. Thus, the interplay between the two timing miRNAs expression played an important role in phase change.

Despite showing a prolonged juvenile phase, *dh* mutant also delayed the heading date (Fig 1A and 1B). Photoperiod is the most important environmental cue for rice flowering [70]. Rice presents two independent photoperiod pathways, one involving *Hd1* and the other involving *Ehd1*, which control heading date by regulating *Hd3a* and *RFT1* [57]. In the *dh* mutant, *Ehd1* and *Hd3a/RFT1* expression was almost completely repressed (Fig 5A–5C). According to a flowering network proposed in rice [57], we found that the expression levels of six genes (*RID1/OsID1/Ehd2*, *OsMADS50*, *OsGI*, *Hd1*, *OsMADS56*, and *Ghd7*), located upstream of *Ehd1* and *Hd3a*, did not differ between ZH11 and the *dh* mutant (Fig 5D–5I). RFT1 and Hd3a proteins were produced in leaves and moved from the leaves to SAM to promote floral transition [57]. However, the expression of *RFT1* and *Hd3a* were completely down-regulated in leaves in *dh* mutant (Fig 5A, 5C). Through this process, the floral meristem identity determined genes located in the SAM cannot accept the flowering signal leading to late-flowering. We also found that the expression of *OsPHYC* was much higher in *dh* mutant, compared to that in ZH11 (S7 Fig). Mutation of either *phyB* or *phyC* causes moderate early flowering under long-day photoperiod in rice [71]. *PhyC* is thought to play an important role for flowering in barley [72]. Therefore, we believe that *OsPHYC* may be influencing flowering in rice. Thus, *osa-miR171c* regulates flowering time by promoting the expression of *OsPHYC* independently of the *Hd1/Ehd1-Hd3a/RFT1* pathway (Fig 5), that is, osa-miR171c works in distinct way in leaves to affect the reproductive transition. These data suggested that osa-miR171c plays important roles in two independent transitions, by repressing the juvenile—adult phase change and the vegetative—reproductive stage transition. By regulating several different genetic pathways, osa-miR171c integrates the phase transitions and may be acts as a master switch regulator of phase transition.

### The osa-miR171c*-OsHAMs* module is required for the maintenance of SAM indeterminacy

Maintenance of an indeterminate state and regulation of stem cell population are fundamental aspects of the SAM function. In *Arabidopsis*, stem cell maintenance is regulated by WUSCHEL-CLAVATA (WUS-CLV) feedback loop genes [27]. In rice, *FON1* (similar to *CLV1*) and *FON4* (similar to *CLV3*) negatively regulate stem cell maintenance [30,73–75], while *WOX4* and *OSH1* promote stem cell identity [76,77]. In *dh* mutant, the shoot apex maintained indeterminacy for a longer period. Three genes (*FON1*, *FON4* and *WOX4*), related with stem cell maintenance, were significantly down-regulated in *dh* mutant, whereas *OSH1* remained unchanged (Fig 6M). This suggests that the WUS-CLV feedback loop pathway was mainly affected in *dh* mutant. The *fon4* mutant had enlarged shoot apex and inflorescence meristem, which causes an increase in culm thickness and primary rachis branch number [74,75]. *dh* mutant also present these phenotypes, which may result from the down-regulation of the *FON4* gene. However, it is unclear what the role of *OsHAMs* is in the WUS-CLV feedback loop pathway. Mutation of *Arabidopsis AtHAM1*,*2*,*3* genes altered the relative expression positions of *WUS* and *CLV3* in SAM [44], and generated supernumerary cell layers between the L1 layer and the organizing centre in SAM [43]. On the other hand, the normal expression of *CLV3* in WT not only relied on the basipetal transport of an L1-derived signal, but also required the acropetal transport of WUS signal [78]. In *dh* mutant, SAM also produced so many supernumerary meristem cell layers, blocking the transport of the L1-derived and WUS signals, and causing the alteration of *FON4* expression pattern. Therefore, we hypothesise that the function of *OsHAMs* is required for normal *OsFON4* expression, similar to *Arabidopsis*. These data suggested that *osa-miR171c-OsHAMs* was involved in the maintenance of SAM indeterminacy by affecting the WUS-CLV3 feedback loop.

Here, we also compared the expression of several genes (*ASP1*, *APO1* and *APO2*) known to be related to primary branches number [59,60,79], because *dh* mutants showed an increase number of primary branches. However, the expression of these genes did not differ between *dh* mutant and ZH11 (Fig 6M). Previous studies have shown that the number of primary branches in rice is determined by the initial size of the reproductive shoot apex [61]. In *dh* mutant, the initial size of the reproductive shoot apex is obviously bigger than that in ZH11 (Fig 6E, 6J and 6K). This is consistent with the increased number of primary branches found in *dh* mutant (Fig 7A–7C).

These results suggested that maintenance of SAM indeterminacy by miR171 among *Arabidopsis*, barley and rice is conserved through the regulation of the WUS-CLV feedback loop [42,45].

### The osa-miR171c*-OsHAMs* module role in the phase transition regulatory pathway

Based on data from *Arabidopsis* and barley, miR171c has been confirmed to play a role in controlling the phase change [42–45]. Here, we proposed that the osa-miR171c-*OsHAMs* module regulates phase change in rice through three regulatory pathways (Fig 8). Firstly, up-regulation of osa-miR171c prolongs vegetative growth and delays the juvenile—adult phase transition by increasing the expression of miR156, similar to barley [45]. Secondly, osa-miR171c and its targets could affect the expression of florigens *RFT1* and *Hd3a* by altering the expression of *OsPHYC*. Thirdly, osa-miR171c and its targets affect SAM maintenance by regulating the homologs of the WUS/CLV3 feedback loop, similar to *Arabidopsis* [44].

## Supporting Information

S1 Fig Molecular analyses of the *dh* T-DNA-inserted mutant. (A) Diagram of the T-DNA-inserted site in the promoter of the *OsMIR171c* gene. The triangle indicates the site of the T-DNA insertion. LB and RB represent the left and right borders of the T-DNA. *Ubiquitin* and *35S* promoter are indicated by the red and blue arrows, respectively. Solid lines represent intergenic regions, while black boxes represent genes near the T-DNA-inserted site. The left border of the T-DNA was facing Os04g0623901. Inserted figure is a close-up view of the T-DNA detail in left, which shows *Ubiquitin* and *35S* promoters and two enhancers in the *35S* promoter. (**B)** Southern blot analysis for the T-DNA-insertion locus number. *Hygromycin* (*Hyg)* gene in the T-DNA insertion was used as probe for hybridization. (**C)** Expression comparison of *OsMIR171c* in the leaves at different developing stages (seedling, tillering, and heading) between ZH11 and *dh* mutant by qRT-PCR. *e-EF-1a* was used as internal control. Data represent three experiments.
(TIF)

S2 Fig PCR identification of heterologous and homologous mutant. (A) Position of primers designed. (B) PCR genotyping of the T_2_ rice plants for the *dh* mutant. Wild-type plants (3 and 9); heterologous-*dh* mutant (1, 4, 6, 7, 8, and 10,); the homologous *dh* mutant (5). Large and the small bands indicate that the products were amplified from the genomic DNA or the T-DNA-insertion genomic DNA, respectively.
(TIF)

S3 Fig Sequence analysis of osa-miR171c. (A) Sequence alignment of rice and *Arabidopsis* miR171 family. (B) Agarose gel analysis of the RT-PCR products, validating the presence of an osa-miR171c long primary transcript in rice. R, root; BP, booting panicle.
(TIF)

S4 Fig Amino acid sequence alignment (A) and phylogenetic tree analysis (B) of HAM proteins. (A) Amino acid sequence alignment of HAM from rice, *Arabidopsis*, and *Petunia*. Sequence alignment was performed using ClustalX1.83. LHRI, VHIID, LHRII, PFYRE, and SAW were the five specific domains belonging to the GRAS family. (B) Phylogenetic tree of HAM proteins; the sequences were retrieved from the NCBI database using OsHAM1 as query. MEGA 4 software was used with the neighbour-joining method using the parameters of p-distance, complete deletion, and bootstrap (1,000 replicates).
(TIF)

S5 Fig Three of the four OsHAMs were located in the nucleus. (A) Amino acid sequence alignment of partial OsHAMs. Three of the OsHAMs had putative nuclear signal sequences (NLS), indicated by the asterisks. (B) Subcellular localization of four OsHAMs in rice protoplasts. Transformed rice protoplasts were first identified by GFP fluorescence (first column) of the OsHAM-GFP fusion proteins, then these cells were checked for chlorophyll auto-fluorescence (second column), corresponding bright-field image (3^rd^ column), and merged image (4^th^ column) of the first and the second column. Free GFP was used as control. Bar = 5 μm.
(TIF)

S6 Fig Anatomical features of ZH11 and a *dh* mutant. (A) SEM images of a cross section of the ZH11 culms. (B) SEM images of a cross section of the *dh* culms. Scale bars = 500 μm.
(TIF)

S7 Fig Expression level of *OsPHYC* in ZH11 and the *dh* mutant. Total RNAs were extracted from leaves, collected from 21-day-old plants grown under short days (SD, 9-h-day/15-h-night), from 21-day-old plants in a field under natural day (ND), and from 30-day-old plants grown under long days (LD, 14-h-day/10-h-night). Values are shown as means of two biological replicates. Error bars indicate standard deviation. *e-EF-1a* was used as internal control.
(TIF)

S8 Fig The *dh* mutant exhibit abnormalities in leaf morphology. (A and B) Distribution of leaf veins in ZH11 (A) and in a *dh* mutant (B). (C and D) SEM observation of leaf surface structures in ZH11 (C) and a *dh* mutant (D). (E) Statistical analysis of mastoideus diameters. Error bar indicates SD from at least 50 measurements. MV, middle vein; LV, large vein; SV, small vein; MA, mastoideus; ST, stoma; DSC, double silica cell. Bars = 500 μm (A, B), 50 μm (C, D). ** represents extreme significant difference comparing to ZH11 (p<0.01)
(TIF)

S1 Table Primers used in this study. (DOC)

S2 Table Putative target genes of osa-miR171c. (DOC)

S3 Table Segregation ratio of the *delayed-heading* phenotype from T_2_ to T_4_ generations of the heterologous *dh* mutant. (DOC)

S4 Table Analysis of the promoter of the *OsMIR171c* gene. (DOC)
