# Supplementary figures and images for: Rice osa-miR171c Mediates Phase Change from Vegetative to Reproductive Development and Shoot Apical Meristem Maintenance by Repressing Four OsHAM Transcription Factors
Source: PLoS One. 2015 May 29;10(5):e0125833. doi: 10.1371/journal.pone.0125833 (PMC4449180; doi:10.1371/journal.pone.0125833)

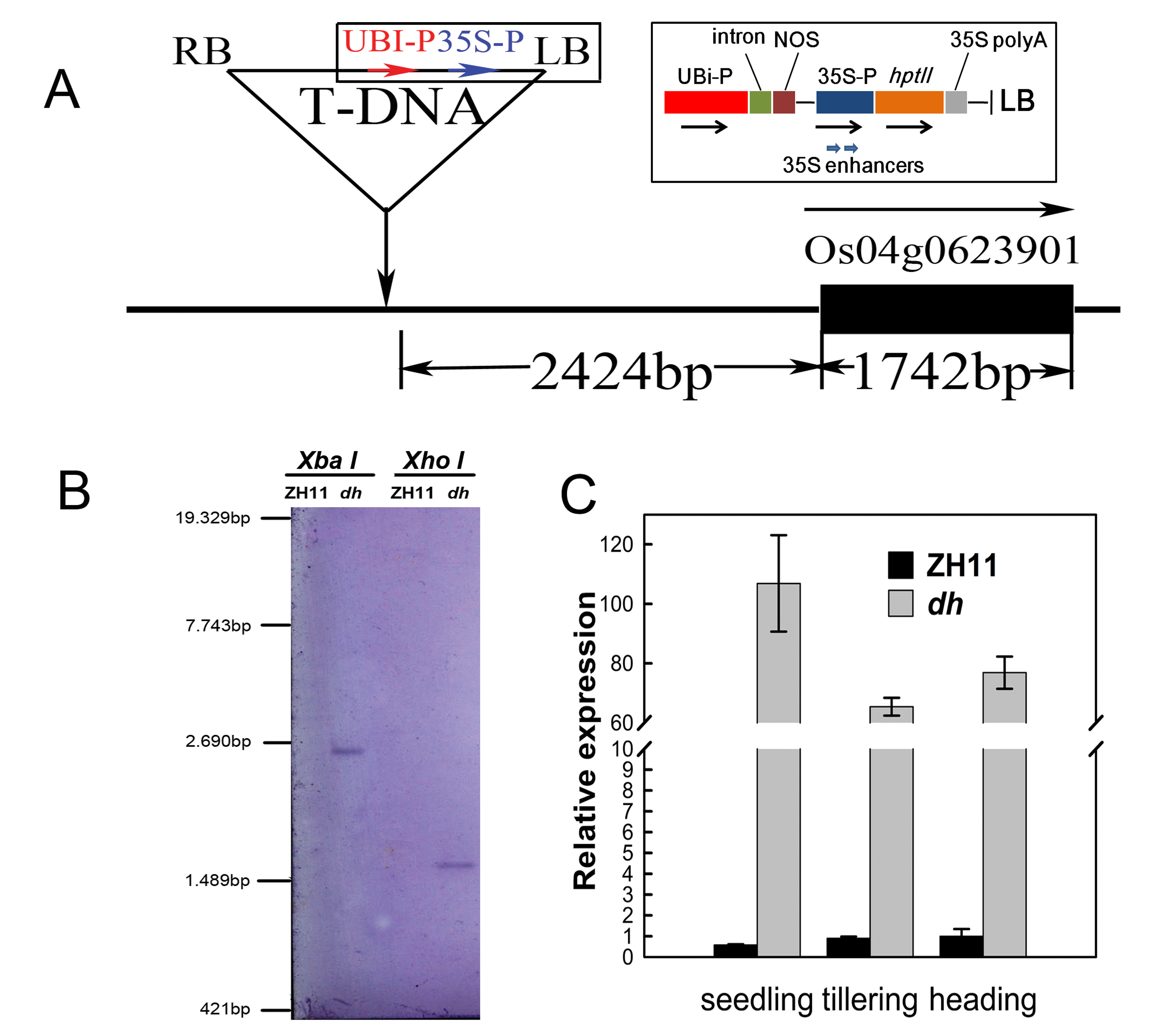

Supplement: S1 Fig — (A) Diagram of the T-DNA-inserted site in the promoter of the OsMIR171c gene. The triangle indicates the site of the T-DNA insertion. LB and RB represent the left and right borders of the T-DNA. Ubiquitin and 35S promoter are indicated by the red and blue arrows, respectively. Solid lines represent intergenic regions, while black boxes represent genes near the T-DNA-inserted site. The left border of the T-DNA was facing Os04g0623901. Inserted figure is a close-up view of the T-DNA detail in left, which shows Ubiquitin and 35S promoters and two enhancers in the 35S promoter. (B) Southern blot analysis for the T-DNA-insertion locus number. Hygromycin (Hyg) gene in the T-DNA insertion was used as probe for hybridization. (C) Expression comparison of OsMIR171c in the leaves at different developing stages (seedling, tillering, and heading) between ZH11 and dh mutant by qRT-PCR. e-EF-1a was used as internal control. Data represent three experiments. (TIF) [file pone.0125833.s001.tif]

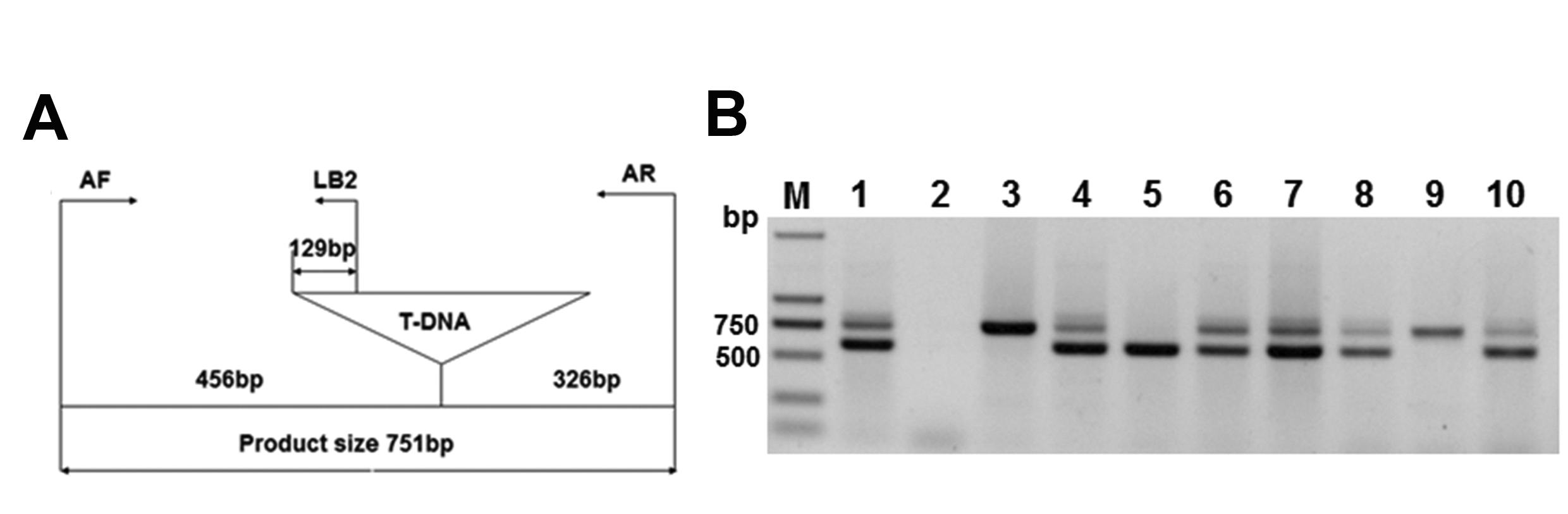

Supplement: S2 Fig — (A) Position of primers designed. (B) PCR genotyping of the T2 rice plants for the dh mutant. Wild-type plants (3 and 9); heterologous-dh mutant (1, 4, 6, 7, 8, and 10,); the homologous dh mutant (5). Large and the small bands indicate that the products were amplified from the genomic DNA or the T-DNA-insertion genomic DNA, respectively. (TIF) [file pone.0125833.s002.tif]

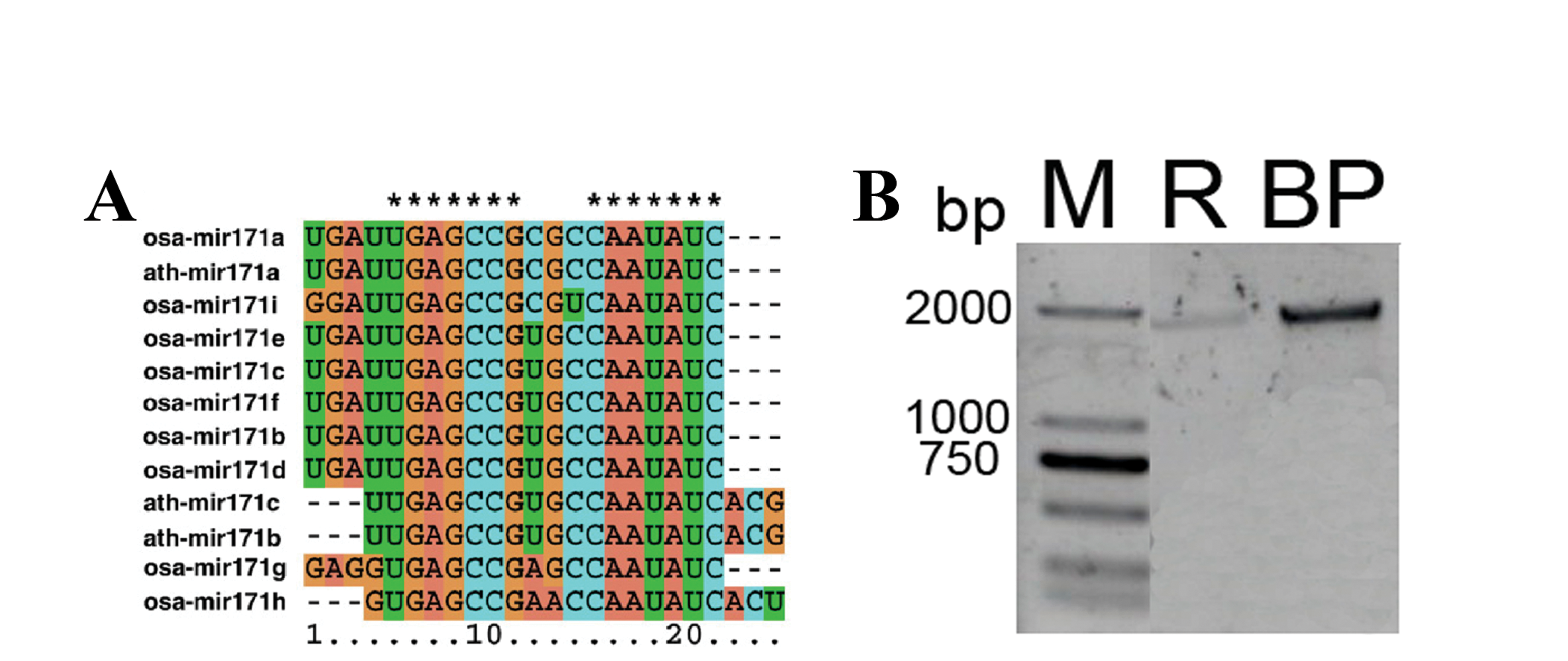

Supplement: S3 Fig — (A) Sequence alignment of rice and Arabidopsis miR171 family. (B) Agarose gel analysis of the RT-PCR products, validating the presence of an osa-miR171c long primary transcript in rice. R, root; BP, booting panicle. (TIF) [file pone.0125833.s003.tif]

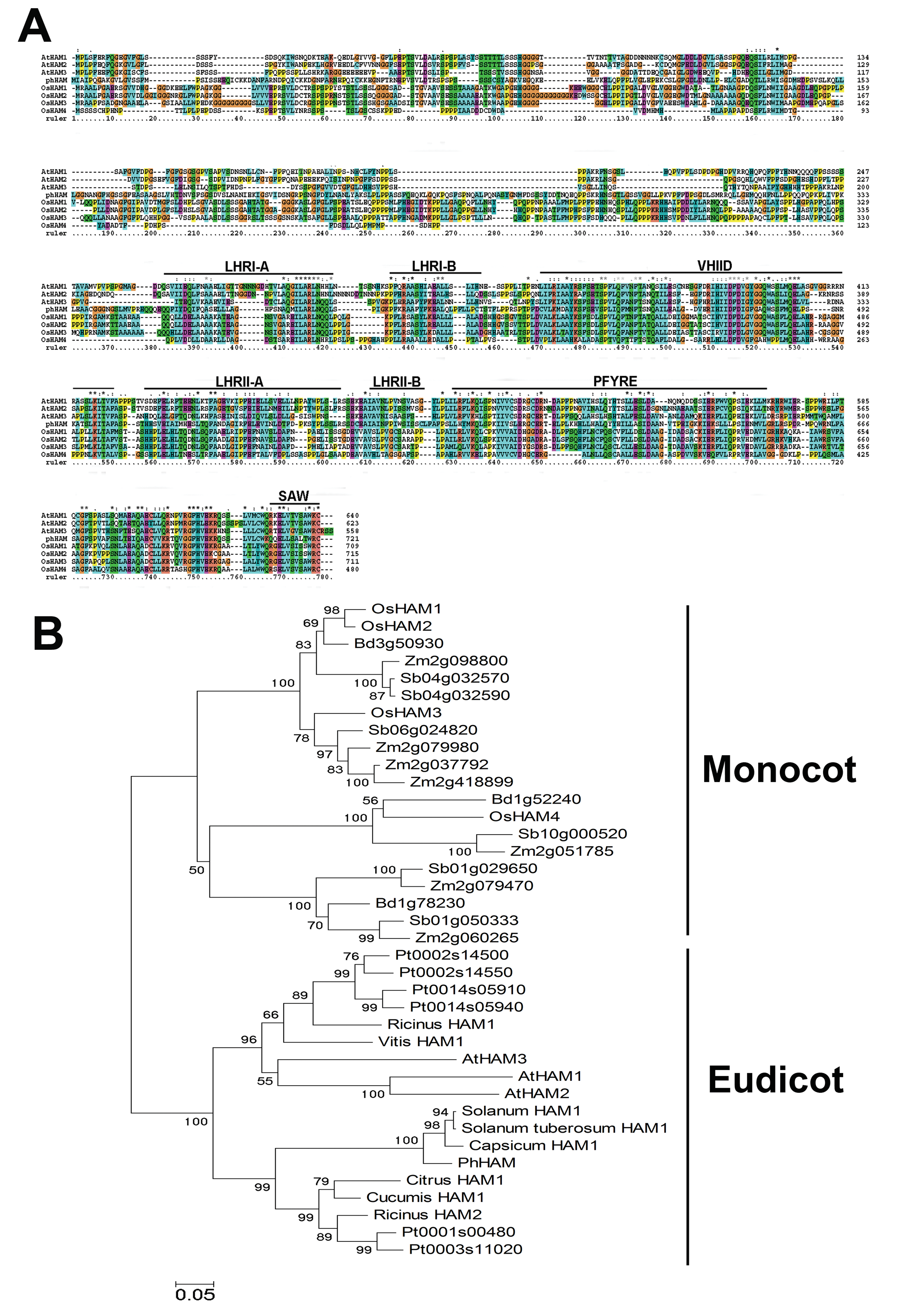

Supplement: S4 Fig — (A) Amino acid sequence alignment of HAM from rice, Arabidopsis, and Petunia. Sequence alignment was performed using ClustalX1.83. LHRI, VHIID, LHRII, PFYRE, and SAW were the five specific domains belonging to the GRAS family. (B) Phylogenetic tree of HAM proteins; the sequences were retrieved from the NCBI database using OsHAM1 as query. MEGA 4 software was used with the neighbour-joining method using the parameters of p-distance, complete deletion, and bootstrap (1,000 replicates). (TIF) [file pone.0125833.s004.tif]

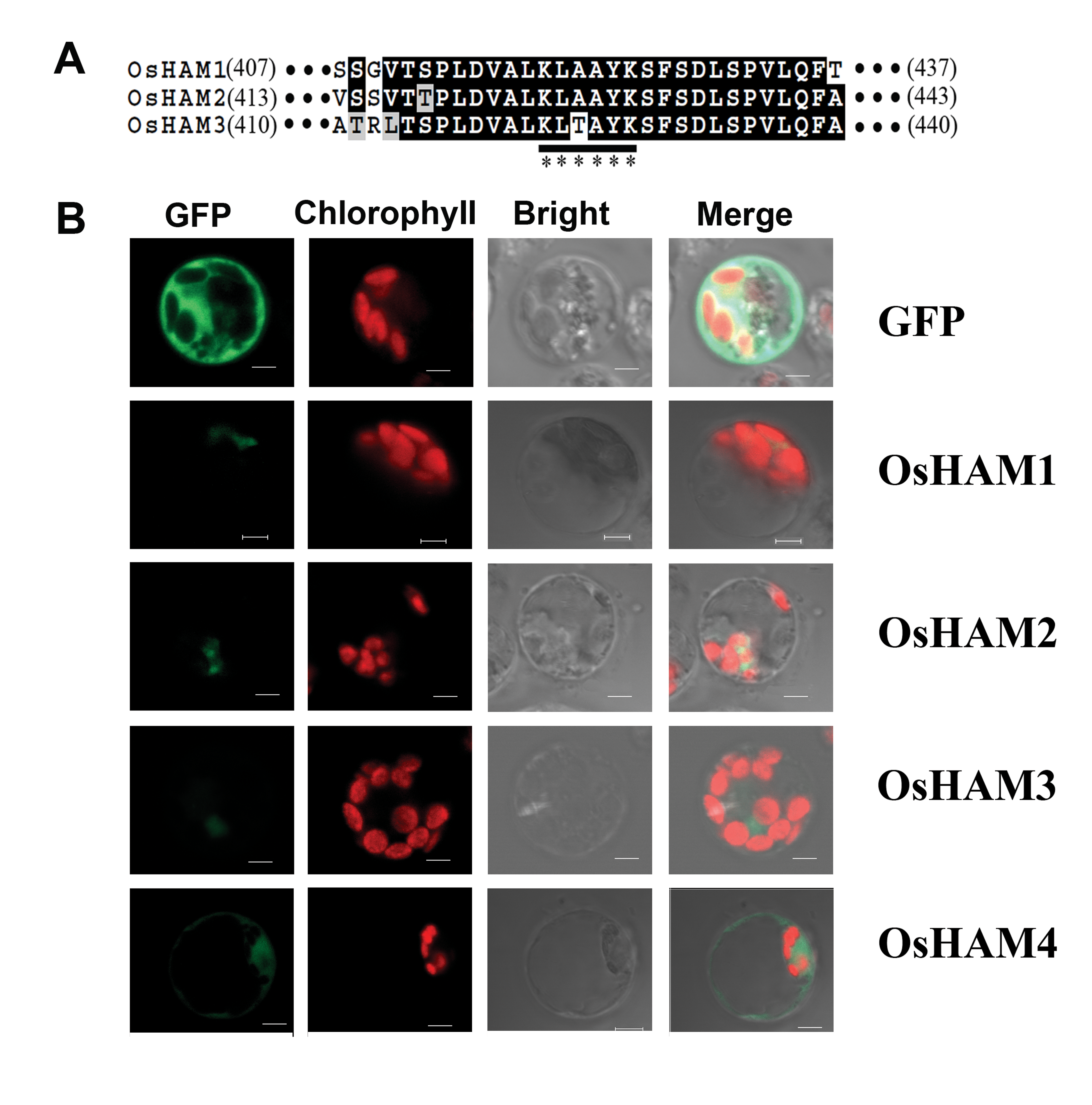

Supplement: S5 Fig — (A) Amino acid sequence alignment of partial OsHAMs. Three of the OsHAMs had putative nuclear signal sequences (NLS), indicated by the asterisks. (B) Subcellular localization of four OsHAMs in rice protoplasts. Transformed rice protoplasts were first identified by GFP fluorescence (first column) of the OsHAM-GFP fusion proteins, then these cells were checked for chlorophyll auto-fluorescence (second column), corresponding bright-field image (3rd column), and merged image (4th column) of the first and the second column. Free GFP was used as control. Bar = 5 μm. (TIF) [file pone.0125833.s005.tif]

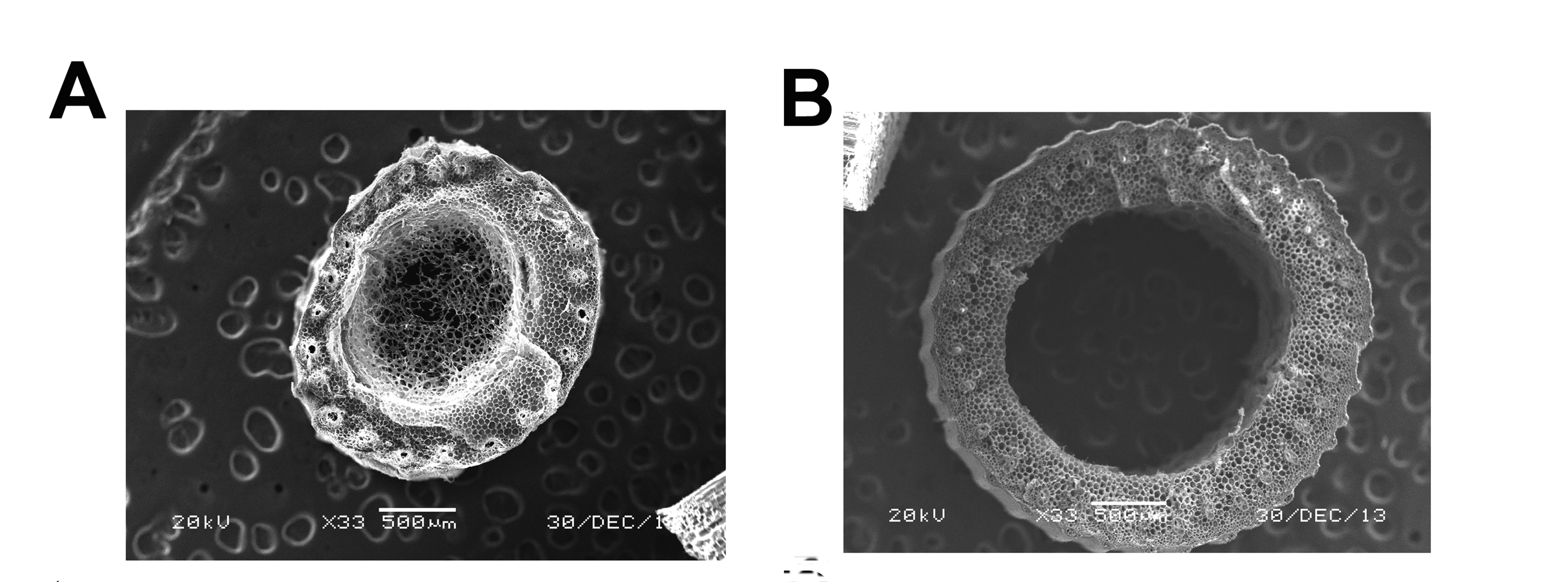

Supplement: S6 Fig — (A) SEM images of a cross section of the ZH11 culms. (B) SEM images of a cross section of the dh culms. Scale bars = 500 μm. (TIF) [file pone.0125833.s006.tif]

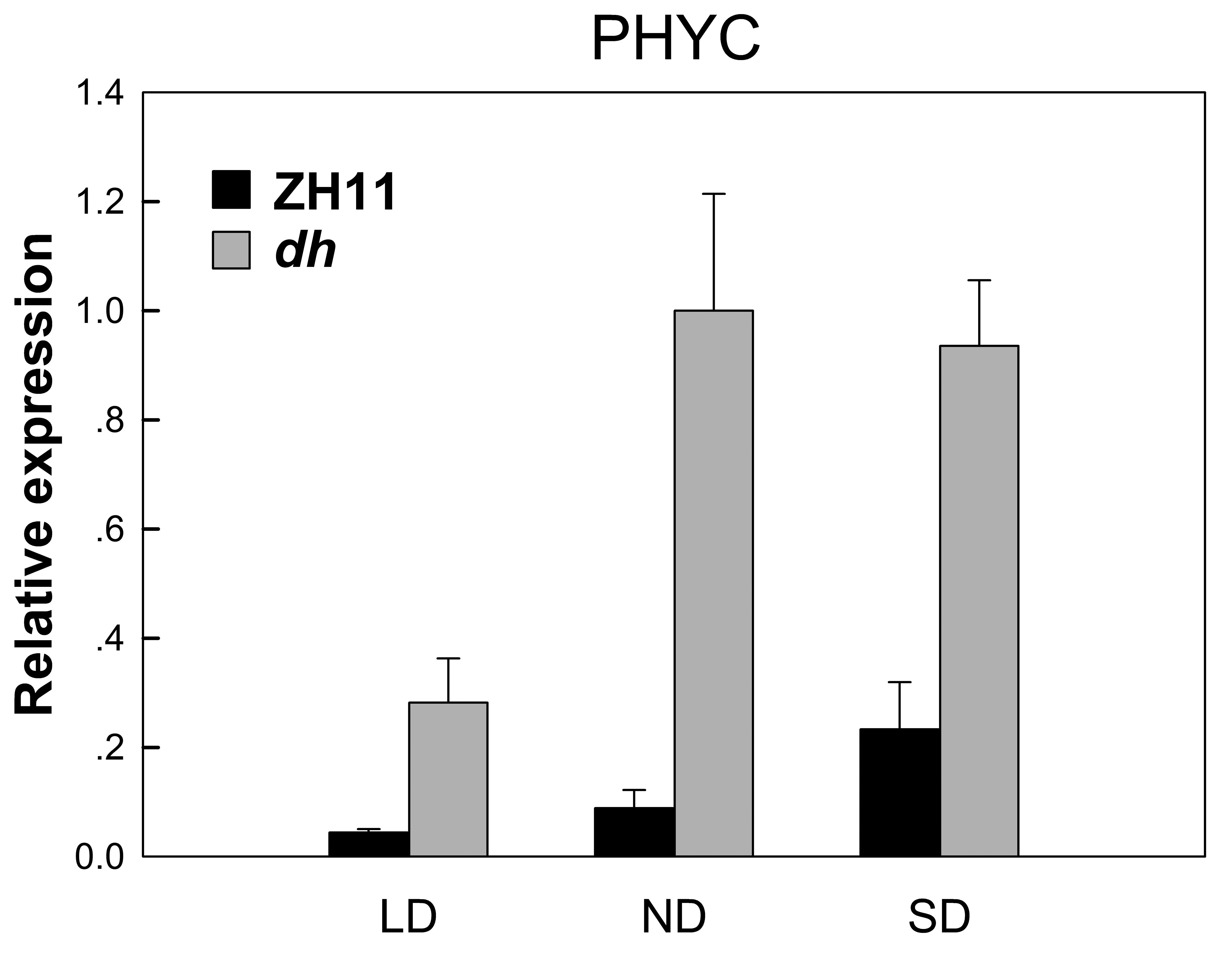

Supplement: S7 Fig — Total RNAs were extracted from leaves, collected from 21-day-old plants grown under short days (SD, 9-h-day/15-h-night), from 21-day-old plants in a field under natural day (ND), and from 30-day-old plants grown under long days (LD, 14-h-day/10-h-night). Values are shown as means of two biological replicates. Error bars indicate standard deviation. e-EF-1a was used as internal control. (TIF) [file pone.0125833.s007.tif]

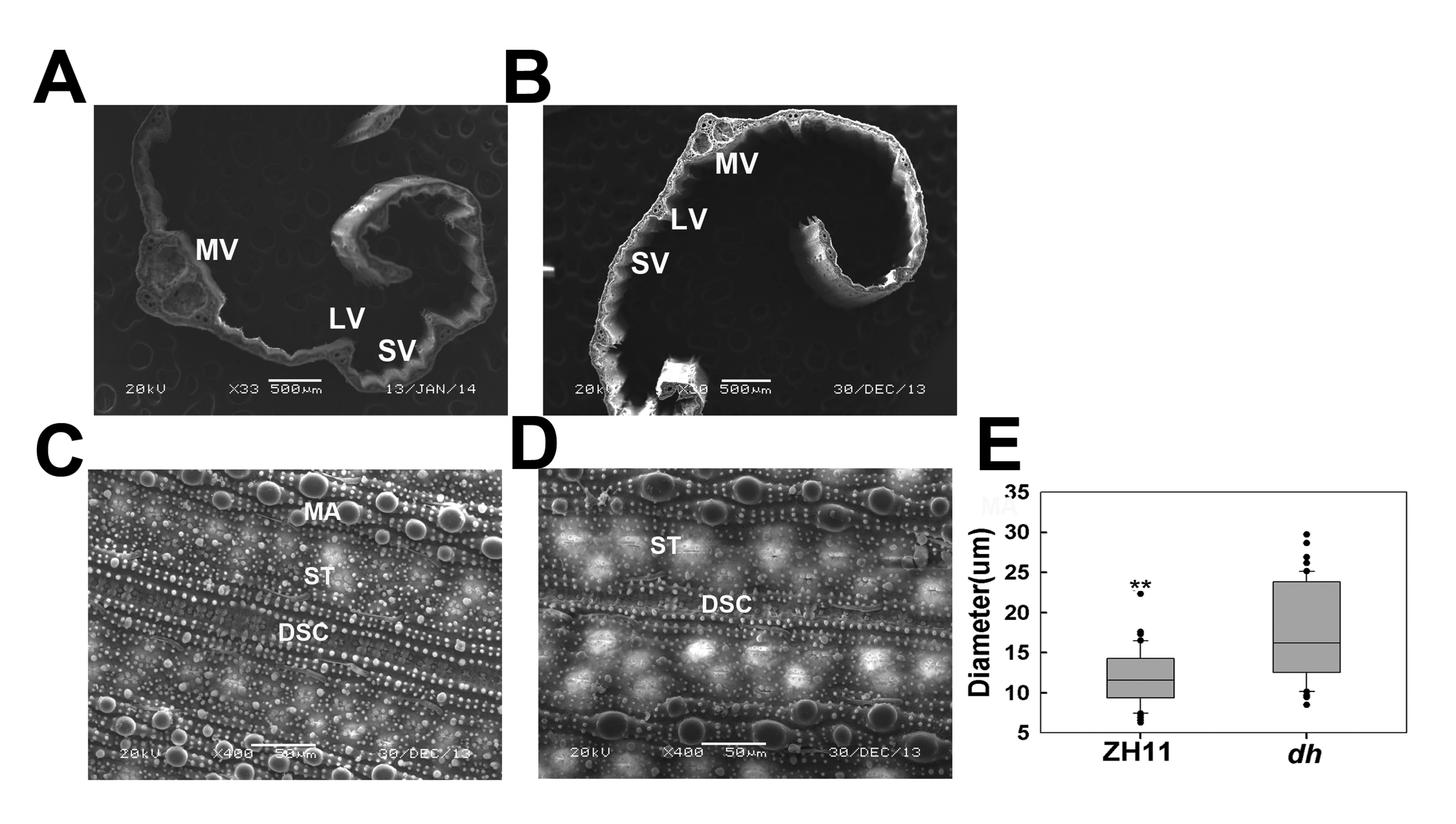

Supplement: S8 Fig — (A and B) Distribution of leaf veins in ZH11 (A) and in a dh mutant (B). (C and D) SEM observation of leaf surface structures in ZH11 (C) and a dh mutant (D). (E) Statistical analysis of mastoideus diameters. Error bar indicates SD from at least 50 measurements. MV, middle vein; LV, large vein; SV, small vein; MA, mastoideus; ST, stoma; DSC, double silica cell. Bars = 500 μm (A, B), 50 μm (C, D). ** represents extreme significant difference comparing to ZH11 (p<0.01) (TIF) [file pone.0125833.s008.tif]
