# Supplementary material for: Rice osa-miR171c Mediates Phase Change from Vegetative to Reproductive Development and Shoot Apical Meristem Maintenance by Repressing Four OsHAM Transcription Factors
Source: PLoS One. 2015 May 29;10(5):e0125833. doi: 10.1371/journal.pone.0125833 (PMC4449180; doi:10.1371/journal.pone.0125833)
Supplement: S1 Table — (DOC) [file pone.0125833.s009.doc]

**S1 Table. List of the primers in this study.**

**1 qRT-PCR primers**

| **Name** | **Sequences(5’-3’)** |
| --- | --- |
| OsMADS50-F | CAGGCCAGGAATAAGCTGGAT |
| OsMADS50-R | TTAGGATGGTTTGGTGTCATTGC |
| eEF-1α-F | GCACGCTCTTCTTGCTTTC |
| eEF-1α-R | AGGGAATCTTGTCAGGGTTG |
| OsMIR171c-F | CCCCACTCTGATCCCATGTCCTC |
| OsMIR171c -R | CCCCATTCTGATTGAACCGCACC |
| RFT1-F | TGACCTAGATTCAAAGTCTAATCCTT |
| RFT1-R | TGCCGGCCATGTCAAATTAATAAC |
| Hd3a-F | GCTCACTATCATCATCCAGCATG |
| Hd3a-R | CCTTGCTCAGCTATTTAATTGCATAA |
| Hd1-F | TCAGCAACAGCATATCTTTCTCATCA |
| Hd1-R | TCTGGAATTTGGCATATCTATCACC |
| Ghd7-F | ATGGGGATGGCCAATGAGGAGTC |
| Ghd7-R | GAGGAATCCGGCCGCCTTTTTTC |
| RID1/Ehd2/OsID1-F | CGACGACAATAGCTCGATCGC |
| RID1/Ehd2/OsID1-R | GTGCATGGTCACGGAGCCTT |
| MADS56-F | ACACATTCCGTTCGCAAATCCGCAC |
| MADS56-R | ACACTCGGGTTCTCAATCCGCTTC |
| GI-F | ATCGTTCTGCAGGCCGAGA |
| GI-R | TCACCAATGCTTCTGGGCTAT |
| Ehd1-F | GGATGCAAGGAAATCATGGA |
| Ehd1-R | AATCCCATCGGAAATCTTGG |
| APO2-F | AGGAAGTGTTGTCCGACGAG |
| APO2-R | CTGCATGTCCAGCCTTAGGT |
| WOX4-F | GCACGCTCGACCCTTCCT |
| WOX4-R | TCTCCGGTGCCTCTTTGG |
| APO1-F | GTCATCTGAGTTGGTAGTGTG |
| APO1-R | CAACAGATCTCATGGCAAG |
| FON4-F | GGTTGCTCTGCTGCTTGTTGCT |
| FON4-R | TGCTTCGTCTTCGGCTCTGTCA |
| FON1-F | GCCTCTGCTACCTCCACCATG |
| FON1-R | ACCACGCTCTTCTCGTCCACTC |
| ASP1-F | TGGACACTGTTTCGGGACAAGC |
| ASP1-R | TGGTAGGAGCGGCACAAGAATG |
| Os02g0662700-F | AGGCAACCGAGGCTGGCAAT |
| Os02g0662700-R | GGAGCACAGGTGACAGGTCAGA |
| Os02g0663100-F | GGCAACCGAGGCTGGCAATT |
| Os02g0663100-R | ATGGTGGCTGTCAGCGAGTG |
| Os04g0555000-F | CTCAGTCGGCAACTCCATAGGC |
| Os04g0555000-R | GGTTGTGCTGGCGATCTCATCC |
| Os06g0105200-F | TATGCCGACGCCGACACCTT |
| Os06g0105200-R | GATGATTGAGCCGCGCCAATATCT |
| Os03g0135600-F | TAGTTAGGTTGGGGCTGGGTGTG |
| Os03g0135600-R | TCGATCACTTCCGTCGGCTTTTCG |
| Os05g0417100-F | TCAGTGCCAGTGACCCAGCCTTATC |
| Os05g0417100-R | GCAGTGCAGTTCAGCGAGCCTTTC |
| Os06g0105300-F | GGGCCAAGTGTACCAGGCAGAG |
| Os06g0105300-R | GCGCAAGGTGGAGCAGTTCGTC |
| Os10g0551200-F | CGGGCGAAGCGGATTTTAGTTAGG |
| Os10g0551200-R | GGGTTCCAACAACACCATTGCGAC |
| U6-F | CGATAAAATTGGAACGATACAGA |
| U6-R | ATTTGGACCATTTCTCGATTTGT |
| MIR156-F | GCGGCGGTGACAGAAGAGAGT |
| Universal R | GTGCAGGGTCCGAGGTATTC |
| MIR1713P-F | TTCCTTGATTGAGCCGTGCC |
| MIR1715P-F | GGCGCTGTTGGCATGGTTC |
| MIR172-F | GCGGCGCAGAATGTTGATGATG |

**2 5`RACE primers**

| **Name** | **Sequences(5’-3’)** |
| --- | --- |
| Os02g0662700-INR | GGATGGTGAGAAGCAGTCG |
| Os02g0662700-OUTR | GCGAATGAGTTCCGCAGGATTG |
| Os06g0105200-INR | GCTGGATGTGAAGGTGGTGAA |
| Os06g0105200-OUTR | CGTGAACTCGAACGGGATG |
| Os02g0663100-INR | AGTGCCTGAGTTGCCGTGA |
| Os02g0663100-OUTR | CCGCGAACAATGGCAAGGTCAC |
| Os04g0555000-INR | GCAGGTCGGAGAAGGATTTGT |
| Os04g0555000-OUTR | ATGAGGCGGCTGAGACGAA |
| Os10g0551200-INR | CGTGAAGACGGTGAAGTGGAA |
| Os10g0551200-OUTR | TAACCGACGGAGACCACCA |

**3 other primers**

| **Name** | **Sequences(5’-3’)** |
| --- | --- |
| M156-RT | GTCGTATCCAGTGCAGGGTCCGAGGTATTCGCACTGGATACGACGTGCTC |
| M171-3P-RT | GTCGTATCCAGTGCAGGGTCCGAGGTATTCGCACTGGATACGACGATATT |
| M171-5P-RT | GTCGTATCCAGTGCAGGGTCCGAGGTATTCGCACTGGATACGACTTTGAT |
| M172-RT | GTCGTATCCAGTGCAGGGTCCGAGGTATTCGCACTGGATACGACATGCA |
| U6-RT | ATTTGGACCATTTCTCGATTTGT |

**Table S2 Putative target genes of osa-miR171c**

| Gene  Name* | Gene ID in MSU | Gene ID in RAP-DB | mRNA | Description | Targeted by osa-miR171c |
| --- | --- | --- | --- | --- | --- |
| *OsHAM1*(*OsGRAS9*) | LOC_Os02g44360 | Os02g0662700 | AK072900 | Similar to Scl1 protein | Yes |
| *OsHAM2*(*OsGRAS8*) | LOC_Os02g44370 | Os02g0663100 | AK101035 | GRAS transcription factor domain containing protein | Yes |
|  | LOC_Os03g04300 | Os03g0135600 | AK241598 | Similar to Ankyrin repeat protein |  |
| *OsHAM3*(*OsGRAS20*) | LOC-Os04g46860 | Os04g0555000 | AK100757 | GRAS transcription factor domain containing protein | Yes |
|  | LOC_Os05g34460 | [Os05g0417100](http://rapdb.dna.affrc.go.jp/viewer/gbrowse_details/build5?name=Os01g0710200) | AK073199 | Peptidase S1C, HrtA/DegP2/Q/S family protein |  |
| *OsGRAS28* | LOC_Os06g01620 | Os06g0105300 | AK106868 | Hypothetical protein |  |
| *OsHAM4* | LOC_Os06g01620 | Os06g0105350 | NO | Similar to Scarecrow-like 6 | Yes |
|  | LOC_Os09g38330 | Os09g0555600 | AK101142 | MORN motif repeat containing protein |  |
| *(OsGRAS37)* | LOC_Os10g40390 | Os10g0551200 | AK106239 | Similar to Scl1 protein |  |

1. The putative target genes are predicated in http://plantgrn.noble.org/psRNATarget/.
2. Description and mRNA accession are obtained from RAP-DB database (http://rapdb.dna.affrc.go.jp/).
3. MSU: <http://rice.plantbiology.msu.edu/index.shtml>
4. * Gene name in bracet from Liu and Widmer (2014) Plant Mol. Biol. Rep. DOI 10.1007/s11105-014-0721-5

**Table S3 The segregation ratio in three generation family**

|  | WT | *dh*(+/-) | *dh* | $\chi_{C}^{2}$ | $\chi_{0.05，1}^{2}$ |
| --- | --- | --- | --- | --- | --- |
| T2 | 13 | 37 | 12 | 0.344 | 3.84 |
| T3 | 3 | 3 | 3 | 0.037 | 3.84 |
| T4 | 41 | 85 | 50 | 0.189 | 3.84 |

**Table S4 Putative cis-acting elements in *OsMIR171c* promoter**

| **Function** | **Site Name** | **Organism** | **Position** | **Strand** | **Sequence** |
| --- | --- | --- | --- | --- | --- |
| **Light responsive** | **ATCT-motif** | ***Zea mays*** | **342** | **+** | **AATCTGATCG** |
|  | **Box 4** | ***Petroselinum crispum*** | **110** | **+** | **ATTAAT** |
|  |  | ***Petroselinum crispum*** | **286** | **+** | **ATTAAT** |
|  | **CATT-motif** | ***Zea mays*** | **304** | **+** | **GCATTC** |
|  |  | ***Zea mays*** | **1464** | **-** | **GCATTC** |
|  |  | ***Zea mays*** | **743** | **+** | **GCATTC** |
|  | **G-Box** | ***Pisum sativum*** | **1076** | **+** | **CACGTT** |
|  | **G-box** | ***Zea mays*** | **77** | **-** | **CACGAC** |
|  |  | ***Zea mays*** | **1076** | **+** | **CACGTT** |
|  | **GAG-motif** | ***Spinacia oleracea*** | **395** | **+** | **AGAGATG** |
|  |  | ***Hordeum vulgare*** | **870** | **-** | **GGAGATG** |
|  |  | ***Spinacia oleracea*** | **573** | **+** | **AGAGATG** |
|  |  | ***Hordeum vulgare*** | **1255** | **-** | **GGAGATG** |
|  | **I-box** | ***Arabidopsis thaliana*** | **24** | **-** | **acGATAATC** |
|  | **Sp1** | ***Zea mays*** | **625** | **-** | **CC(G/A)CCC** |
|  |  | ***Zea mays*** | **1037** | **+** | **CC(G/A)CCC** |
|  |  | ***Zea mays*** | **677** | **-** | **CC(G/A)CCC** |
|  |  | ***Zea mays*** | **1038** | **+** | **CC(G/A)CCC** |
|  |  | ***Zea mays*** | **676** | **-** | **CC(G/A)CCC** |
|  |  | ***Zea mays*** | **680** | **-** | **CC(G/A)CCC** |
|  | **TCCC-motif** | ***Spinacia oleracea*** | **669** | **-** | **TCTCCCT** |
|  |  | ***Spinacia oleracea*** | **1231** | **+** | **TCTCCCT** |
|  |  | ***Spinacia oleracea*** | **1171** | **+** | **TCTCCCT** |
| **Defense** | **ARE** | ***Zea mays*** | **1282** | **-** | **TGGTTT** |
| **Meristem expression** | **CAT-box** | ***Arabidopsis thaliana*** | **792** | **+** | **GCCACT** |
| **Endosperm expression** | **GCN4_motif** | ***Oryza sativa*** | **748** | **+** | **CAAGCCA** |
|  | **Skn-1_motif** | ***Oryza sativa*** | **1067** | **+** | **GTCAT** |
| **Zein metabolism** | **O2-site** | ***Zea mays*** | **1351** | **-** | **GATGACATGG** |
| **MYBHv1 binding** | **CCAAT-box** | ***Hordeum vulgare*** | **1018** | **-** | **CAACGG** |
| **MeJA-responsiveness** | **CGTCA-motif** | ***Hordeum vulgare*** | **156** | **+** | **CGTCA** |
|  |  | ***Hordeum vulgare*** | **882** | **-** | **CGTCA** |
|  |  | ***Hordeum vulgare*** | **537** | **+** | **CGTCA** |
|  |  | ***Hordeum vulgare*** | **437** | **+** | **CGTCA** |
|  |  | ***Hordeum vulgare*** | **550** | **-** | **CGTCA** |
|  | **TGACG-motif** | ***Hordeum vulgare*** | **156** | **-** | **TGACG** |
|  |  | ***Hordeum vulgare*** | **882** | **+** | **TGACG** |
|  |  | ***Hordeum vulgare*** | **537** | **-** | **TGACG** |
|  |  | ***Hordeum vulgare*** | **437** | **-** | **TGACG** |
|  |  | ***Hordeum vulgare*** | **550** | **+** | **TGACG** |
| **Auxin-responsive** | **TGA-element** | ***Brassica oleracea*** | **441** | **+** | **AACGAC** |
|  |  | ***Brassica oleracea*** | **823** | **-** | **AACGAC** |
|  |  | ***Brassica oleracea*** | **497** | **+** | **AACGAC** |
| **Salicylic acid response** | **TCA-element** | ***Brassica oleracea*** | **1148** | **-** | **GAGAAGAATA** |
|  |  | ***Brassica oleracea*** | **1247** | **-** | **GAGAAGAATA** |
|  |  |  |  |  |  |
