# Supplementary material for: Rice osa-miR171c Mediates Phase Change from Vegetative to Reproductive Development and Shoot Apical Meristem Maintenance by Repressing Four OsHAM Transcription Factors
Source: PLoS One. 2015 May 29;10(5):e0125833. doi: 10.1371/journal.pone.0125833 (PMC4449180; doi:10.1371/journal.pone.0125833)
Supplement: S2 Table — (DOC) [file pone.0125833.s010.doc]

**S2 Table. Putative target genes of osa-miR171c**

| Gene  Name* | Gene ID in MSU | Gene ID in RAP-DB | mRNA | Description | Targeted by osa-miR171c |
| --- | --- | --- | --- | --- | --- |
| *OsHAM1*(*OsGRAS9*) | LOC_Os02g44360 | Os02g0662700 | AK072900 | Similar to Scl1 protein | Yes |
| *OsHAM2*(*OsGRAS8*) | LOC_Os02g44370 | Os02g0663100 | AK101035 | GRAS transcription factor domain containing protein | Yes |
|  | LOC_Os03g04300 | Os03g0135600 | AK241598 | Similar to Ankyrin repeat protein |  |
| *OsHAM3*(*OsGRAS20*) | LOC-Os04g46860 | Os04g0555000 | AK100757 | GRAS transcription factor domain containing protein | Yes |
|  | LOC_Os05g34460 | [Os05g0417100](http://rapdb.dna.affrc.go.jp/viewer/gbrowse_details/build5?name=Os01g0710200) | AK073199 | Peptidase S1C, HrtA/DegP2/Q/S family protein |  |
| OsGRAS28 | LOC_Os06g01620 | Os06g0105300 | AK106868 | Hypothetical protein |  |
| *OsHAM4* | LOC_Os06g01620 | Os06g0105350 | NO | Similar to Scarecrow-like 6 | Yes |
|  | LOC_Os09g38330 | Os09g0555600 | AK101142 | MORN motif repeat containing protein |  |
| (OsGRAS37) | LOC_Os10g40390 | Os10g0551200 | AK106239 | Similar to Scl1 protein |  |

1. The putative target genes are predicated in http://plantgrn.noble.org/psRNATarget/.
2. Description and mRNA accession are obtained from RAP-DB database (http://rapdb.dna.affrc.go.jp/).
3. MSU: <http://rice.plantbiology.msu.edu/index.shtml>
4. * Gene name in bracet from Liu and Widmer (2014) Plant Mol. Biol. Rep. DOI 10.1007/s11105-014-0721-5
