# Supplementary material for: Rice osa-miR171c Mediates Phase Change from Vegetative to Reproductive Development and Shoot Apical Meristem Maintenance by Repressing Four OsHAM Transcription Factors
Source: PLoS One. 2015 May 29;10(5):e0125833. doi: 10.1371/journal.pone.0125833 (PMC4449180; doi:10.1371/journal.pone.0125833)
Supplement: S3 Table — (DOC) [file pone.0125833.s011.doc]

**S3 Table. The segregation ratio in three generation family**

|  | WT | *dh*(+/-) | *dh* | $\chi_{C}^{2}$ | $\chi_{0.05，1}^{2}$ |
| --- | --- | --- | --- | --- | --- |
| T2 | 13 | 37 | 12 | 0.344 | 3.84 |
| T3 | 3 | 3 | 3 | 0.037 | 3.84 |
| T4 | 41 | 85 | 50 | 0.189 | 3.84 |
