# Supplementary material for: Rice osa-miR171c Mediates Phase Change from Vegetative to Reproductive Development and Shoot Apical Meristem Maintenance by Repressing Four OsHAM Transcription Factors
Source: PLoS One. 2015 May 29;10(5):e0125833. doi: 10.1371/journal.pone.0125833 (PMC4449180; doi:10.1371/journal.pone.0125833)
Supplement: S4 Table — (DOC) [file pone.0125833.s012.doc]

**S4 Table. Putative cis-acting elements in *OsMIR171c* promoter**

| **Function** | **Site Name** | **Organism** | **Position** | **Strand** | **Sequence** |
| --- | --- | --- | --- | --- | --- |
| **Light responsive** | **ATCT-motif** | ***Zea mays*** | **342** | **+** | **AATCTGATCG** |
|  | **Box 4** | ***Petroselinum crispum*** | **110** | **+** | **ATTAAT** |
|  |  | ***Petroselinum crispum*** | **286** | **+** | **ATTAAT** |
|  | **CATT-motif** | ***Zea mays*** | **304** | **+** | **GCATTC** |
|  |  | ***Zea mays*** | **1464** | **-** | **GCATTC** |
|  |  | ***Zea mays*** | **743** | **+** | **GCATTC** |
|  | **G-Box** | ***Pisum sativum*** | **1076** | **+** | **CACGTT** |
|  | **G-box** | ***Zea mays*** | **77** | **-** | **CACGAC** |
|  |  | ***Zea mays*** | **1076** | **+** | **CACGTT** |
|  | **GAG-motif** | ***Spinacia oleracea*** | **395** | **+** | **AGAGATG** |
|  |  | ***Hordeum vulgare*** | **870** | **-** | **GGAGATG** |
|  |  | ***Spinacia oleracea*** | **573** | **+** | **AGAGATG** |
|  |  | ***Hordeum vulgare*** | **1255** | **-** | **GGAGATG** |
|  | **I-box** | ***Arabidopsis thaliana*** | **24** | **-** | **acGATAATC** |
|  | **Sp1** | ***Zea mays*** | **625** | **-** | **CC(G/A)CCC** |
|  |  | ***Zea mays*** | **1037** | **+** | **CC(G/A)CCC** |
|  |  | ***Zea mays*** | **677** | **-** | **CC(G/A)CCC** |
|  |  | ***Zea mays*** | **1038** | **+** | **CC(G/A)CCC** |
|  |  | ***Zea mays*** | **676** | **-** | **CC(G/A)CCC** |
|  |  | ***Zea mays*** | **680** | **-** | **CC(G/A)CCC** |
|  | **TCCC-motif** | ***Spinacia oleracea*** | **669** | **-** | **TCTCCCT** |
|  |  | ***Spinacia oleracea*** | **1231** | **+** | **TCTCCCT** |
|  |  | ***Spinacia oleracea*** | **1171** | **+** | **TCTCCCT** |
| **Defense** | **ARE** | ***Zea mays*** | **1282** | **-** | **TGGTTT** |
| **Meristem expression** | **CAT-box** | ***Arabidopsis thaliana*** | **792** | **+** | **GCCACT** |
| **Endosperm expression** | **GCN4_motif** | ***Oryza sativa*** | **748** | **+** | **CAAGCCA** |
|  | **Skn-1_motif** | ***Oryza sativa*** | **1067** | **+** | **GTCAT** |
| **Zein metabolism** | **O2-site** | ***Zea mays*** | **1351** | **-** | **GATGACATGG** |
| **MYBHv1 binding** | **CCAAT-box** | ***Hordeum vulgare*** | **1018** | **-** | **CAACGG** |
| **MeJA-responsiveness** | **CGTCA-motif** | ***Hordeum vulgare*** | **156** | **+** | **CGTCA** |
|  |  | ***Hordeum vulgare*** | **882** | **-** | **CGTCA** |
|  |  | ***Hordeum vulgare*** | **537** | **+** | **CGTCA** |
|  |  | ***Hordeum vulgare*** | **437** | **+** | **CGTCA** |
|  |  | ***Hordeum vulgare*** | **550** | **-** | **CGTCA** |
|  | **TGACG-motif** | ***Hordeum vulgare*** | **156** | **-** | **TGACG** |
|  |  | ***Hordeum vulgare*** | **882** | **+** | **TGACG** |
|  |  | ***Hordeum vulgare*** | **537** | **-** | **TGACG** |
|  |  | ***Hordeum vulgare*** | **437** | **-** | **TGACG** |
|  |  | ***Hordeum vulgare*** | **550** | **+** | **TGACG** |
| **Auxin-responsive** | **TGA-element** | ***Brassica oleracea*** | **441** | **+** | **AACGAC** |
|  |  | ***Brassica oleracea*** | **823** | **-** | **AACGAC** |
|  |  | ***Brassica oleracea*** | **497** | **+** | **AACGAC** |
| **Salicylic acid response** | **TCA-element** | ***Brassica oleracea*** | **1148** | **-** | **GAGAAGAATA** |
|  |  | ***Brassica oleracea*** | **1247** | **-** | **GAGAAGAATA** |
|  |  |  |  |  |  |
